# Structural and biochemical characterization of cauliflower mosaic virus reverse transcriptase

**DOI:** 10.1016/j.jbc.2024.107555

**Published:** 2024-07-11

**Authors:** Chandrasekaran Prabaharan, Małgorzata Figiel, Roman H. Szczepanowski, Krzysztof Skowronek, Weronika Zajko, Vinuchakkaravarthy Thangaraj, Sebastian Chamera, Elżbieta Nowak, Marcin Nowotny

**Affiliations:** 1 Laboratory of Protein Structure, International Institute of Molecular and Cell Biology in Warsaw, Warsaw, Poland; 2 Biophysics and Bioanalytics Facility, International Institute of Molecular and Cell Biology in Warsaw, Warsaw, Poland

**Keywords:** cauliflower mosaic virus (CaMV), reverse transcriptases, X-ray crystallography, protein nucleic acid complexes, Caulimoviridae, plant viruses

## Abstract

Reverse transcriptases (RTs) are enzymes with DNA polymerase and RNase H activities. They convert ssRNA into dsDNA and are key enzymes for the replication of retroviruses and retroelements. Caulimoviridae is a major family of plant-infecting viruses. Caulimoviruses have a circular dsDNA genome that is replicated by reverse transcription, but in contrast to retroviruses, they lack integrase. Caulimoviruses are related to Ty3 retroelements. Ty3 RT has been extensively studied structurally and biochemically, but corresponding information for caulimoviral RTs is unavailable. In the present study, we report the first crystal structure of cauliflower mosaic virus (CaMV) RT in complex with a duplex made of RNA and DNA strands (RNA/DNA hybrid). CaMV RT forms a monomeric complex with the hybrid, unlike Ty3 RT, which does so as a dimer. Results of the RNA-dependent DNA polymerase and DNA-dependent DNA polymerase activity assays showed that individual CaMV RT molecules are able to perform full polymerase functions. However, our analyses showed that an additional CaMV RT molecule needs to transiently associate with a polymerase-competent RT molecule to execute RNase H cuts of the RNA strand. Collectively, our results provide details into the structure and function of CaMV RT and describe how the enzyme compares to other related RTs.

Reverse transcriptases (RTs) play a critical role in the life cycle of retroviruses, retrotransposons, and some DNA viruses. These enzymes catalyze the process of reverse transcription (the conversion of ssRNA into dsDNA). RTs exhibit two distinct activities: (*i*) DNA polymerase synthesizes DNA using DNA or RNA as a template, and (*ii*) RNase H cleaves the RNA strand of an RNA/DNA heteroduplex during the replication process (1, 2). Retroviruses and long terminal repeat (LTR) retroelements are related, and both replicate with a reverse transcription step and integrate their replicated copies into the genome of the host. Retroviruses form infectious particles that can propagate to other cells, whereas the replication of retrotransposons is restricted to the host cell (1, 3).

Reverse-transcribing elements/viruses belong to the order *Ortervirales* and are divided into three families of retroelements (*Belpaoviridae*, *Metaviridae* [Ty3/Gypsy elements], and *Pseudoviridae* [Ty1/Copia elements]) and two families of viruses (Retroviridae and Caulimoviridae, the latter being closely related to *Metaviridae*) (4). Retroviruses store their genetic information in the form of ssRNA. Caulimoviruses, which infect plants, are pararetroviruses; they have an open-circular dsDNA genome that upon replication is not integrated into the host genome but instead is maintained in the cell in an episomal form (5). Caulimoviruses share these properties with the other family of pararetroviruses, hepadnaviruses (2).

The catalytic activities of all RTs are similar, but they exhibit significant differences in their architecture, subunit composition, and structure. For example, during the evolution of retroviruses from Ty3 retroelements, the RT gained a new RNase H domain that is attached to the C terminus of the protein, while the original RNase H domain lost its catalytic activity to become a connection domain (6, 7). To date, structures of RTs from six retroelement/retrovirus families have been reported. Reverse transcriptases from lentiviruses (HIV-1 (8), feline immunodeficiency virus (9), and human endogenous retrovirus-K (10)) act as heterodimers that consist of a large subunit that contains polymerase and RNase H domains and a small subunit that adopts a different conformation and lacks the RNase H domain. The larger subunit harbors both polymerase and RNase H activities, whereas the smaller subunit plays only a structural role (8, 9, 10). In contrast, RTs from the gammaretroviruses xenotropic murine leukemia-related virus (XMRV) and Moloney murine leukemia virus are active as monomers (11, 12). The RT of *Saccharomyces cerevisiae* retroelement Ty3 forms a nucleic acid substrate-induced asymmetric homodimer (6). Retroviruses from the *Spumaretrovirinae* subfamily (foamy viruses) have a dsDNA genome and replicate using a mechanism that is similar to pararetroviruses (13, 14, 15, 16). The marmoset foamy virus (MFV) RT interacts with RNA/DNA as a monomer, but on dsDNA, it forms a substrate-induced homodimer (14). The mechanisms of action of RTs from various retroviruses/retroelements are remarkably different, and more modes of RT action are expected to be discovered as further RTs are studied.

Caulimoviruses primarily infect plants through aphid transmission. The International Committee on Taxonomy of Viruses classifies Caulimoviridae in six genera: *Caulimoviruses*, *Soymoviruses*, *Cavemoviruses*, *Tungroviruses*, *Badnaviruses*, and *Petuviruses* (17). Caulimoviruses evolved from LTR retroelements (18) but lack LTRs. Their protease-RT-RNase H genomic architecture and replication mechanisms are similar to LTR retroelements (19, 20), but they have a capsid protein instead of a Gag protein and engage in a unique interaction with the vector protein for their vector-mediated transmission (21). Caulimovirus species have specific ORFs that encode both structural and nonstructural proteins (movement protein, protease, RT, RNase H, and transactivator protein) and other proteins whose function is not yet known (19).

Cauliflower mosaic virus (CaMV) is the most studied virus in the Caulimoviridae family. It uses reverse transcriptase as an essential component of its replication process (22, 23). Its replication cycle has two stages: one in the nucleus and the other in the cytoplasm. Once the viral particle enters the plant cell, the viral dsDNA genome is transported to the nucleus with the help of its coat protein (24). The viral dsDNA associates with histones leading to the formation of minichromosomes. Retroviruses have integrase enzymes that integrate their genome into the host genome, but the genome of the caulimoviruses is present as multiple copies of minichromosomes (25, 26). These minichromosomes are transcribed by the host transcription machinery. The two major transcribed RNA products, 35S RNA and 19S RNA, and the minor 8S RNA are transported to the cytoplasm. The 35S RNA functions as a pregenomic RNA, the 19S RNA encodes the P6 gene for the regulation of translation, and the 8S is thought to play a role in RNA silencing. The 35S RNA is reverse transcribed into genomic dsDNA and packaged into new virions. The virions are transported by aphids to other cells or plants (27, 28, 29, 30, 31).

CaMV mainly infects plants from the Brassicaceae family, including radish, turnip, rape, mustard, cauliflower, broccoli, and cabbage. Some CaMV strains infect Solanaceae species and tobacco plants (*Nicotiana* genus) (32, 33). The pioneering transgenic 35S promoter was initially identified from CaMV and has been instrumental in driving transgene expression in genetically modified plants. The 35S promoter or its modified versions are present in approximately 60% of the transgenic crops globally, underscoring its immense significance in plant and agricultural biotechnology (30).

CaMV RT plays a critical role in replication of the virus, but few biochemical studies of the enzyme have been performed (34), with no structural information available for CaMV RT. We determined a structure of CaMV RT in complex with a hybrid substrate complex at 2.35 Å resolution. Structural and biochemical studies showed that the enzyme interacts with hybrid and dsDNA substrates as a monomer, but transient dimer formation is required for RNase H activity.

## Results

### Overall structure

**Figure 1:**
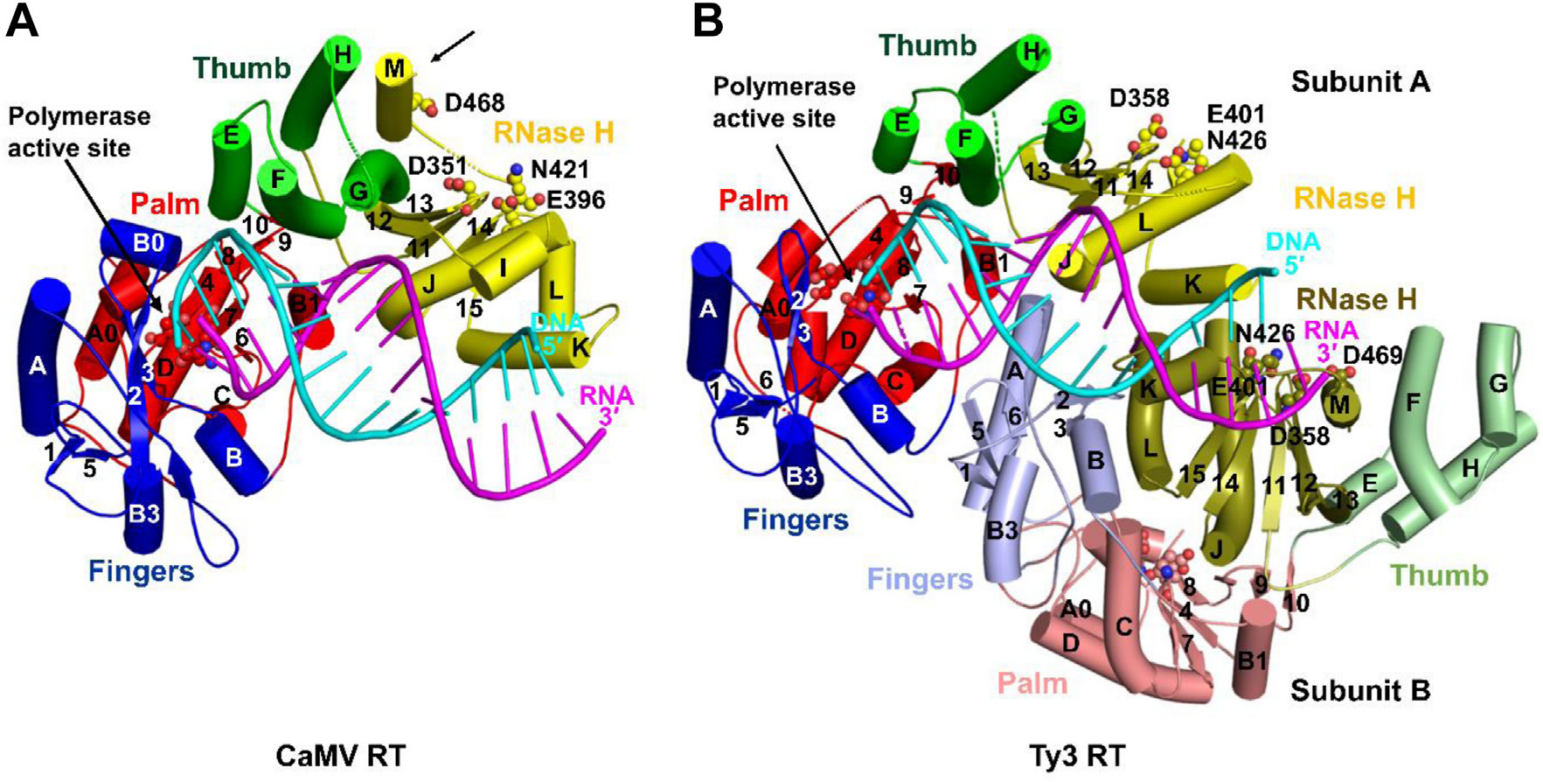
**Cartoon representation of substrate complexes of CaMV RT and Ty3 RT.***A*, CaMV RT structure (present study). Subdomains are colored *blue* for fingers, *red* for palm, *green* for thumb, and *yellow* for RNase H. The RNA strand is shown in *magenta*, and the DNA strand is shown in *cyan*. The α-helices are labeled A to M, and the β-sheets are labeled 1 to 15. The arrow indicates the M-helix of the RNase H domain, displaced from its canonical position in the RNase H fold. *B*, Ty3 RT complex structure (PDB ID: 4OL8). Subunit A is colored and labeled as for CaMV RT. Subunit B is shown in lighter colors, with the RNase H domain in *olive*. Polymerase and RNase H domain catalytic residues are shown as *spheres*.

To elucidate the mechanism of action of CaMV RT, we sought to solve its structure and characterize it biochemically. The protein was efficiently expressed in bacteria as a SUMO fusion protein. It was purified using NiNTA affinity chromatography, and the SUMO tag was removed. Further purification using gel filtration resulted in high-quality protein preparations. The protein in complex with various RNA/DNA hybrids underwent extensive crystallization trials. The best crystals were obtained in the presence of an RNA/DNA hybrid with a 16 bp double-stranded part and a 2-nucleotide (nt) 5′ overhang in the RNA strand (for the sequence, see Experimental procedures). These crystals belonged to the P2_1_2_1_2 space group and diffracted X-rays to a resolution of 2.35 Å at a synchrotron source. The complex structure was solved by the molecular replacement method using a model of CaMV RT protein generated by AlphaFold (35). The data collection and refinement statistics are shown in Table 1. In the structure, the CaMV RT interacts with the hybrid as a monomer (Fig. 1*A*). The electron density was observed for both polymerase (POL) and RNase H domains as well as for all nucleotides of the RNA/DNA hybrid except the 5′ terminal nucleotide of the RNA strand. Sample electron density maps are shown in Fig. S1 and crystal packing interactions are shown in Fig. S2.

**Table 1:** Data collection and refinement statistics for CaMV RT–RNA/DNA complex crystal structure

Parameter	CaMV RT in complex with an RNA/DNA hybrid
Data collection	
Space group	P2_1_2_1_2
Cell dimensions	
a, b, c (Å)	80.8, 140.5, 52.5
α, β, γ (°)	90.0, 90.0, 90.0
Resolution (Å)^a^	45.25-2.35 (2.49-2.35)
R_merge_^a^	0.107 (1.53)
I/σI^a^	9.95 (1.08)
CC_1/2_^a^^b^	0.997 (0.496)
Completeness (%)^a^	99.8 (99.7)
Redundancy^a^	6.0 (5.8)
Refinement	
Resolution (Å)	45.25-2.35
No. of reflections	28163
R_work_ (%)	21.4
R_free_ (%)	26.3
No. of atoms	
Protein	3655
Ligand/ion	675
Water	74
B factors (Å^2^)	
Protein	58.6
Ligand/ion	70.6
Water	57.8
RMSDs	
Bond lengths (Å)	0.008
Bond angles (°)	1.010

The data collection statistics are based on a single crystal.

a Values in parentheses are for the highest-resolution shell.

b CC_1/2_, correlation coefficient between the average intensities in two parts of the unmerged data, each with a random half of the measurements of each unique reflection (57).

CaMV RT is structurally very similar to chain A of Ty3 RT (6) (Protein Data Bank [PDB] ID: 4OL8) with RMSD value after superposition of the two structures equal to 2.0 Å over 334 pairs of Cα atoms (Fig. 1, *A* and *B*). It is also similar to the polymerase-connection part of the p66 chain of HIV-1 RT (8) (PDB ID: 1RTD; RMSD value: 3.5 Å over 309 pairs of Cα atoms). The POL domain of CaMV RT consists of three subdomains: fingers (residues 36–115 and 150–170), palm (residues 1–35, 116–149, and 171–259), and thumb (residues 260–332). The polymerase active site, located in the palm subdomain, is highly conserved and very similar to other RTs (Fig. S3). A unique feature of CaMV RT is a short α-helix in the fingers subdomain (residues 91–97) between β strands 2 and 3 (for the labeling of secondary structure elements, see Figs. 1*A* and S4). It forms interactions with the 3′ end of the DNA primer, which have not been observed in any other RT structure (see below). The thumb subdomain of CaMV RT is very similar to its counterpart in Ty3 RT, but all helices are longer than in Ty3 RT (Fig. 1*B*).

**Figure 2:**
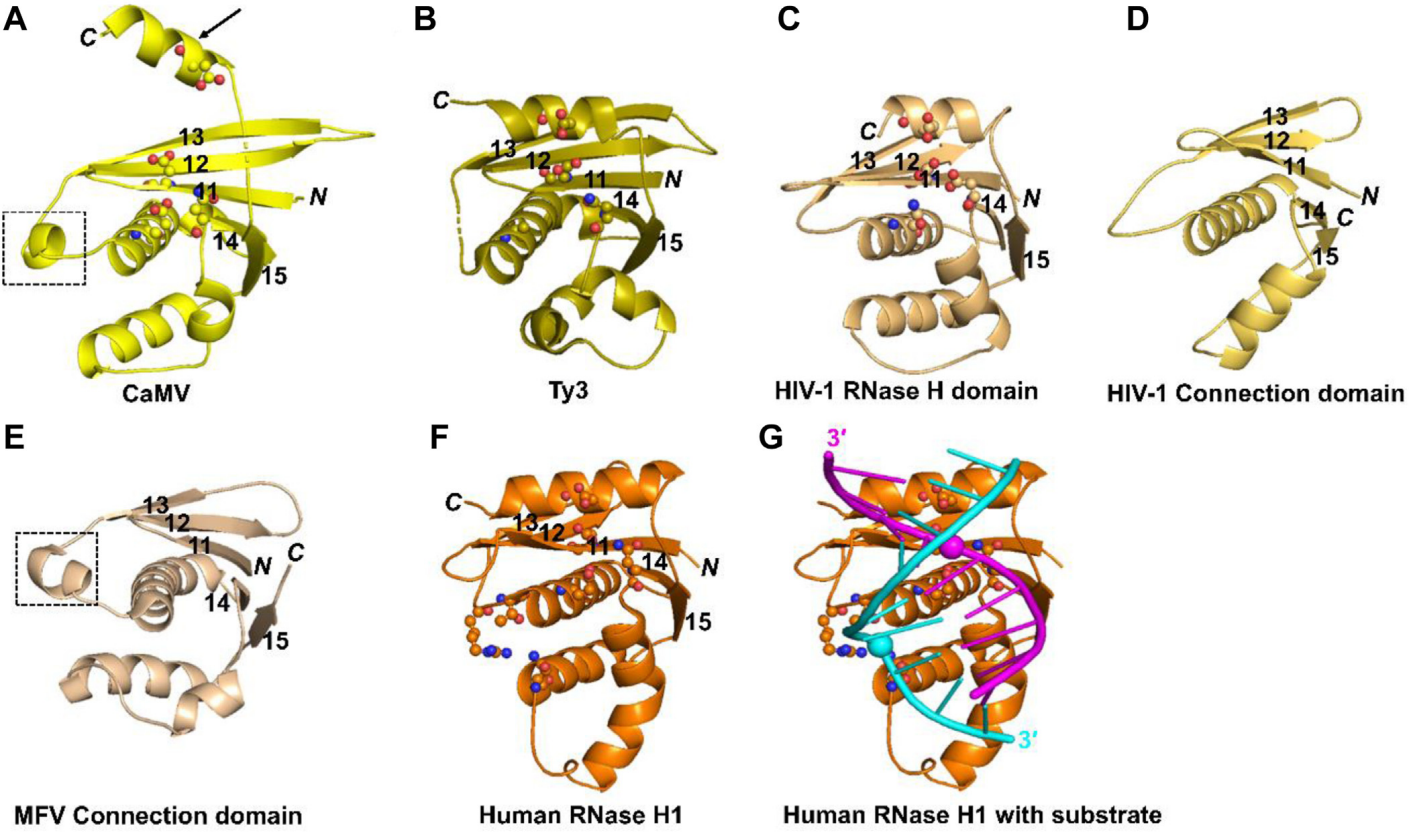
**Comparison of the RNase H domains/protein and connection domains.***A*, cartoon representation of CaMV RNase H domain. *B*, Ty3 RNase H domain (PDB ID: 4OL8) (6). *C*, HIV-1 RNase H domain (PDB ID: 1RTD) (8). *D*, HIV-1 connection domain (PDB ID: 1RTD) (8). *E*, MFV connection domain (PDB ID: 7O0G) (14). *F*, human RNase H1 (protein chain from PDB ID: 2QK9) (37). *G*, human RNase H1 with bound RNA/DNA substrate. The hybrid substrate is displayed as a ladder, *magenta* for the RNA strand and *cyan* for the DNA strand. The active site residues of the RNase H domains/protein and the phosphate binding pocket for the human enzyme are shown as *spheres-and-sticks*. The *spheres* represent phosphate groups that interact with the active site (RNA) and phosphate-binding pocket (DNA). The *arrow* indicates the RNase H domain M-helix displaced from its canonical position in the RNase H fold. The dotted box indicates the short helix (I) between β3 strand and α1-helix in the CaMV RH domain and MFV connection domain.

The RNase H domain (residues 346–474) is linked to the polymerase domain *via* a short linker (residues 333–345). It has a typical RNase H fold with a central β-sheet that is formed by five strands and surrounded by five α-helices (Fig. 2). The central β-sheet is composed of two long antiparallel strands (β11 and β12), and the remaining three short strands are parallel to the first strand (β11). Compared with other cellular and viral RTs, the central β12 and β13 strands of CaMV RT are longer (Fig. S5). Clear electron density was observed for helices I and L, which were not visible in chain A of the Ty3 RT RNase H domain. Helix I is absent in retroviral RNase H domains but conserved in some retroviral connection domains, supporting the idea that during retroviral RT evolution, the original RNase H domain was converted to an inactive connection domain (7).

An important feature of the CaMV RT structure is that α-helix M, which harbors the last residue of the RNase H active site (Asp468), is located further away from the core of the RNase H fold, and this position is stabilized by interactions with the thumb subdomain (Fig. 1). This results in the displacement of Asp468 from its canonical position in the RNase H active site, so the conformation of the RNase H domain in our structure corresponds to an inactivated state.

The blunt end of the RNA/DNA forms crystal contacts with the helix M of the symmetry-related molecule, in particular through residues His465, Phe466, and Phe469 (Fig. S2*B*). This implies that crystal contacts may stabilize the interaction of helix M with the thumb subdomain, which we interpreted as a mechanism of disorganization and inactivation of the RNase H active site. However, further support for the observed arrangement of helix M comes from the fact that in the AlphaFold 3 (36) model of the CaMV RT-RNA/DNA complex, this helix also does not adopt the conformation conducive to active site formation (although it is positioned slightly further away from the thumb subdomain than in the crystal structure). Furthermore, in the structure of the Ty3 RT dimer (PDB ID: 4OL8), for the subunit that adopts the polymerase configuration, no density for the corresponding helix was visible, so the proper formation of the RNase H active site was not observed there either. These considerations support the mechanism in which removal of the last helix of the RNase H domain disorganizes the active site of this domain and inactivates it in the polymerase configuration of CaMV RT.

Superposition of the RNase H domain of CaMV RT on the RNase H domain of chain B of Ty3 RT (RMSD = 2.1 Å over 86 Cα atoms) and human RNase H1 (RMSD = 2.1 Å over 56 Cα atoms) showed high similarity among the three structures (Fig. 2). The RNase H domain of HIV RT is the most different. The RMSD for the superposition of CaMV and HIV-1 RNase H domains is 4.9 Å over the 68 Cα atoms. The structure of CaMV RNase H is also very similar to connection domains of XMRV (RMSD = 1.8 Å over 94 Cα atoms) and MFV (RMSD = 1.1 Å over 73 Cα atoms). However, the CaMV RT RNase H domain is less similar to the HIV connection domain (RMSD = 4.0 Å over 59 Cα atoms) (8, 11, 14).

We note that only a subset of RNase H residues was used for the superposition described above. This is because in the CaMV RT RNase H domain, helix M is displaced from the canonical position in the RNase H fold and helix K is longer than in Ty3 and human RNase H1. Another important difference is that the β12 and β13 strands are also extended by more than 10 residues than other RTs (Fig. S5). Importantly, however, the overall fold and position of the catalytic residues (Asp351, Glu396, Asp421, and Asp468 in CaMV RT) are conserved among all RNase H domains/proteins (6, 8, 37) (Fig. S6).

### Substrate binding

**Figure 3:**
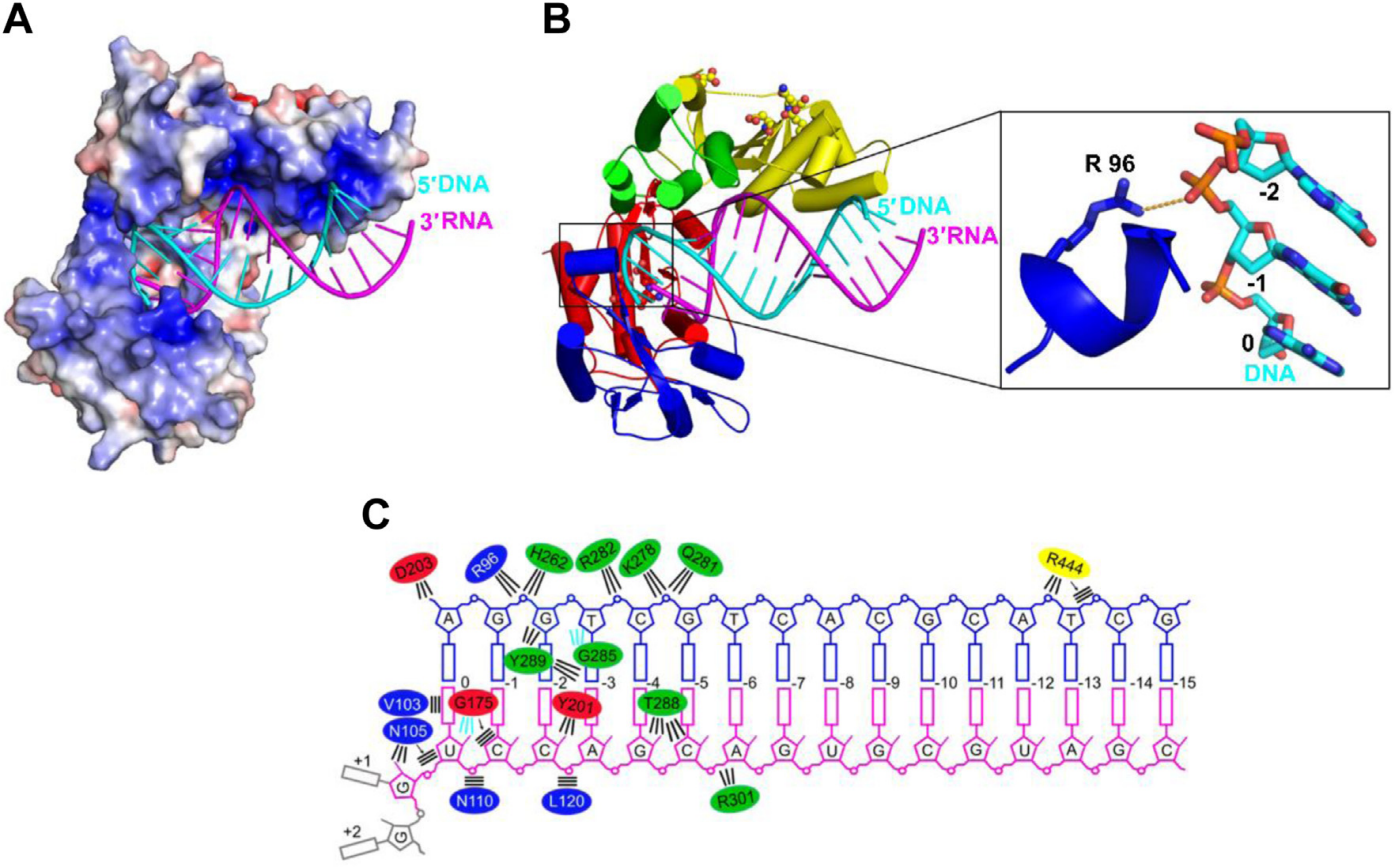
**Substrate binding by CaMV RT.***A*, electrostatic surface potential of CaMV RT from the RT-hybrid complex structure, calculated using the Adaptive Poisson-Boltzman solver plug-in in PyMol (scale bar −5 kTe^−1^ to +5 kTe^−1^; *red*, negative; *blue*, positive). *B*, cartoon representation of CaMV RT. The inset shows a close-up view of the B0 helix that interacts with the phosphate backbone of the 3′ end of the DNA primer. *C*, schematic representation of protein–RNA/DNA interactions. RNA is shown in *magenta*. DNA is shown in *blue*. The nucleotide that is present in the substrate but not observed in the structure is shown in *gray*. *Oval* colors correspond to domain coloring: *blue* for fingers, *red* for palm, *green* for thumb, and *yellow* for RNase H. Radiant lines represent polar contacts. *Parallel lines* represent van der Waals contacts. *Black* lines represent contacts with side chains. *Cyan* lines represent contacts with the protein backbone.

The RNA/DNA substrate in the CaMV RT–RNA/DNA complex adopts a position and conformation that is largely similar to the one that was previously observed for Ty3 RT (Figs. 1, 3). The RNA/DNA duplex is mostly in an A-form conformation. Analysis of the electrostatic surface potential of the CaMV RT–hybrid complex structure revealed several positively charged regions, particularly in the DNA primer–binding interface and around the RNase H active site (Fig. 3*A*). One particular feature of CaMV RT is a positively charged region that binds the 3′-OH group of nucleotide −1 (penultimate) of the DNA primer.

Interactions between CaMV RT and RNA/DNA are schematically shown in Figure 3*C*. Nearly all contacts are mediated by the polymerase domain and involve nucleotides +1 to −6 of the RNA/DNA hybrid. There are two contacts from the RNase H domain with DNA nucleotides −12 and −13 (Fig. 3*C*). In viral RTs, the template nucleotide +1 that base pairs with the incoming dNTP is stabilized by a flexible loop that consists of glycine residues, one of which forms a hydrogen bond with the 2′-OH group of RNA. Although CaMV RT has the same loop region, the conserved Gly175 residue interacts with the 2′-OH group at position 0 of the RNA strand. In Ty3 RT, additional stabilization of the +1 template nucleotide in the RNA strand is provided by residues Asp116 and Arg118. These residues are conserved in caulimoviral RTs as positively charged residues. The Asn105 side chain forms an interaction with the 2′-OH group of the +1 template nucleotide of the RNA strand, and Lys107 has no interaction with any nucleotide but is close to nucleotide 0.

The position of the primer end relative to the polymerase active site residues (Asp140, Asp203, and Asp204) is similar between various RTs and the CaMV enzyme (38), showing that our structure represents a polymerase configuration. The CaMV POL catalytic site has a flexible loop region that comprises Phe174, which stabilizes the base pair that is formed by the incoming dNTP. It is similar to the Ty3 POL catalytic site where Phe185 plays a similar role (6). The phosphate backbone of primer DNAs is additionally stabilized by the side chain of residue Arg96 from the B0 helix that forms contacts with nucleotide −1 (Fig. 3, *B* and *C*). Such interactions are not observed in any of the other RTs for which structures have been determined. This could lead to greater stabilization of the primer strand and bring it closer to a catalytic location.

When our CaMV RT–hybrid complex structure was compared with the HIV RT-RNA/DNA-dATP ternary complex (PDB ID: 4PQU), we found that the position of the last nucleotide of the primer in the CaMV RT structure corresponds to the position of the incoming nucleotide from the HIV RT structure (Fig. S3*B*). Thus, the CaMV RT–hybrid complex adopts a pretranslocation state that is different from the post-translocation state in the reference HIV-1 RT structures (PDB ID: 1RTD and 4PQU). The trajectory of the primer in our structure is also different from that observed in the 1RTD and 4PQU structures and the Ty3 RT structure (Fig. S3, *C* and *D*). Interestingly, the primer trajectory in our structure is similar to the structure of HIV-1 RT in complex with dsRNA in the pretranslocation state (PDB ID: 6HAK) (39). As with any crystal structure, we cannot completely exclude the influence of crystal contacts. In particular, the thumb subdomain and the interacting helix M form crystal contacts with the blunt end of the RNA/DNA of the symmetry-related complex molecule. This could alter the position of the thumb subdomain and the trajectory of the DNA strand near the polymerase active site. However, AlphaFold 3 (36) predicts an almost identical position of the thumb subdomain as observed in our structure, so it is likely that the crystal contacts do not perturb this position. Thus, we favor the explanation that the observed structural differences are inherent to the pretranslocation state we have captured in our structure. Importantly, the primer trajectory observed in our CaMV RT structure can be stabilized by a unique short α-helix containing Arg96 (Fig. 3*B*), which stabilizes the 3′ end of the primer. Currently, no pretranslocation state structure of HIV-1 in complex with dsDNA or RNA/DNA with incorporated natural nucleotide is available, making direct comparisons difficult. Further structural and molecular dynamics studies should clarify this issue.

Nucleotides −1 to −4 of the primer strand form contacts between their phosphodiester backbone and thumb subdomain residues (His262, Lys278, Gln281, and Arg282). Gly285 interacts with O4 of the sugar ring of nucleotide −3, and the Tyr289 side chain interacts with the O4 of the sugar ring of nucleotide −2 and nitrogen base of nucleotide −4. The finger domain residue Val103 forms van der Waals contacts with the nitrogen base of nucleotide 0 of the RNA strand. Asn110 and Leu120 form interactions with the phosphodiester backbone of nucleotides −1 and −3 of the RNA strand. The palm domain Gly175 and Tyr201 residues and thumb domain Thr288 residue interact with 2′-OH groups of the RNA. The thumb domain Arg301 residue interacts with the sugar ring backbone of the RNA strand. The CaMV RT also forms more extensive interactions with the primer DNA strand compared with previously studied RTs (XMRV RT, MFV RT, or Ty3 RT).

### Oligomeric state of CaMV RT in solution

In our structure, CaMV RT interacts with the RNA/DNA substrate as a monomer, unlike the homologous Ty3 RT, which functions as a dimer (6). To confirm that this is not a result of our inability to capture the dimeric state in the structure and to check whether the same monomeric form is also observed in solution, we performed gel filtration chromatography experiments using different molar ratios of CaMV RT and the hybrid substrate (1:1, 2:1, and 1:2; Fig. S7). In each case, the mixture of protein and substrate was incubated on ice for 30 min, and then the sample was subjected to gel filtration chromatography. The protein alone was eluted as a single peak with an elution volume, which based on column calibration, corresponded to a molecular weight of 60 kDa (theoretical MW of CaMV RT is 55.8 kDa). The protein–hybrid complex was also eluted as a single peak with a slightly smaller elution volume than the protein alone which corresponded to MW of 75 kDa (theoretical MW of CaMV RT–RNA/DNA complex is 66.5 kDa). Importantly, no peak was present at the elution volume corresponding to the free RNA/DNA hybrid and the observed peak showed higher absorbance at a 260 nm wavelength, which confirmed that it comprised nucleic acid. All these observations indicate the formation of a stable CaMV RT–RNA/DNA complex (Fig. S7). At a molar ratio of 1:1 protein:substrate, no excess hybrid or protein was eluted. For the 2:1 molar ratio, in addition to the complex peak, a small hump, corresponding to excess protein, was also observed. The 1:2 molar ratio experiment also showed clear complex formation, and excess hybrid substrate was eluted in the hybrid elution volume. These results indicate that CaMV RT forms a one-to-one complex with the hybrid.

To test formation of the CaMV RT–dsDNA complex, we performed the same gel filtration experiment with different ratios of CaMV RT and dsDNA (1:1, 2:1, and 1:2). In each case, the mixture of protein and substrate was incubated on ice for 30 min, and then the sample was subjected to gel filtration chromatography. The protein and dsDNA were also analyzed separately. At the molar ratio of 1:1 protein:dsDNA, the first peak eluted earlier than the peaks for protein and dsDNA, and the second peak eluted at an elution volume slightly smaller than that for the dsDNA. The reason for this shift is not clear but we assume this peak corresponds to the excess of dsDNA which did not form the complex with protein. The first eluted peak had higher absorbance at a 260 nm wavelength which showed that it corresponded to CaMV RT in complex with dsDNA. However, the presence of free dsDNA indicated that this complex was unstable. Based on column calibration, the elution volume for the first complex peak corresponded to a molecular mass of 75 kDa. For the 2:1 molar ratio, all of the dsDNA substrate was bound by the enzyme, and a small hump, corresponding to excess protein, was observed at the protein elution volume. The 1:2 molar ratio also showed clear complex formation, but a portion of the substrate eluted in unbound form. These results showed that CaMV RT forms a complex with dsDNA at a 1:1 ratio, but the interaction is weak.

**Figure 4:**
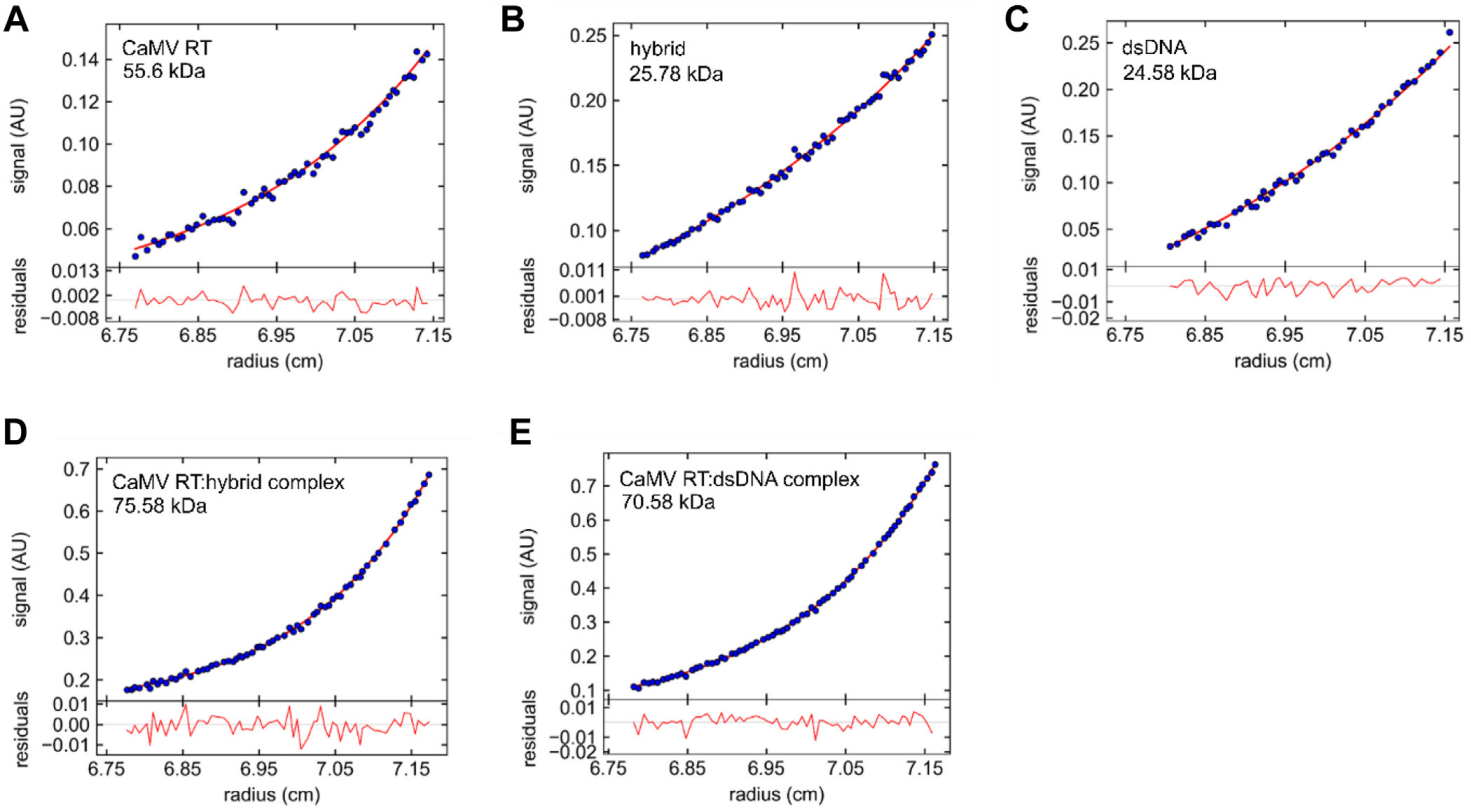
**Sedimentation equilibrium distributions of analytical ultracentrifugation studies.** Sedimentation profiles for (*A*) 2 μM (0.12 mg/ml) CaMV RT, (*B*) 2 μM RNA/DNA hybrid, (*C*) 2 μM dsDNA, (*D*) CaMV RT-hybrid complex at a 1:1 ratio, and (*E*) CaMV RT-dsDNA complex at a 1:1 ratio. All samples were subjected to centrifugation at 10,000 rpm. MWs determined in each experiment are given in each panel.

To further study substrate binding by CaMV RT, the sedimentation equilibrium method was used to characterize the interaction between nucleic acid substrates (RNA/DNA and dsDNA) and the CaMV RT protein. This method allows the precise measurement of masses of both individual components and their complexes and thus determines their mutual interaction. An important advantage of this technique is that, unlike the AUC sedimentation velocity approach, sedimentation equilibrium considers only the mass of molecules, and the different shape of the complex does not affect measurement precision. During the centrifugation of CaMV RT, the RNA/DNA hybrid, and dsDNA (all at a concentration of 2 μM), they existed as monomers, and their experimental masses were very close to theoretical masses that were calculated from amino acid and nucleotide sequences (*e.g.* experimentally determined mass for CaMV RT is 55.6 kDa, as compared to the theoretical mass of 55.8 kDa). At this stage of the analysis, the partial specific volume, typical for such molecules and equal to 0.530 ml/g or 0.540 ml/g, was used for nucleic acids. However, the apparent masses of nucleic acids that were calculated using the v-bar of protein (0.7429 ml/g) were used to analyze masses of the complexes. In the case of both complexes, the results could only be reliably fitted by assuming the presence of a protein–nucleic acid complex that comprises one molecule of protein and nucleic acid (Fig. 4).

In conclusion, gel filtration and analytical centrifugation experiments confirmed that CaMV RT interacts with both RNA/DNA and dsDNA as a monomer.

### Polymerase activity assays

We next wanted to demonstrate the DNA polymerase activity of CaMV RT. We performed RNA- and DNA-dependent DNA polymerase assays. We used 75 nt RNA or DNA templates combined with a 5′-fluorescently labeled 18 nt DNA primer that was complementary to the 3′ terminus of the template (for sequences, see Experimental procedures), and we analyzed reaction products by urea-PAGE with fluorescence detection. We also included the Ty3 RT and PFV RT in our assays for comparison.

**Figure 5:**
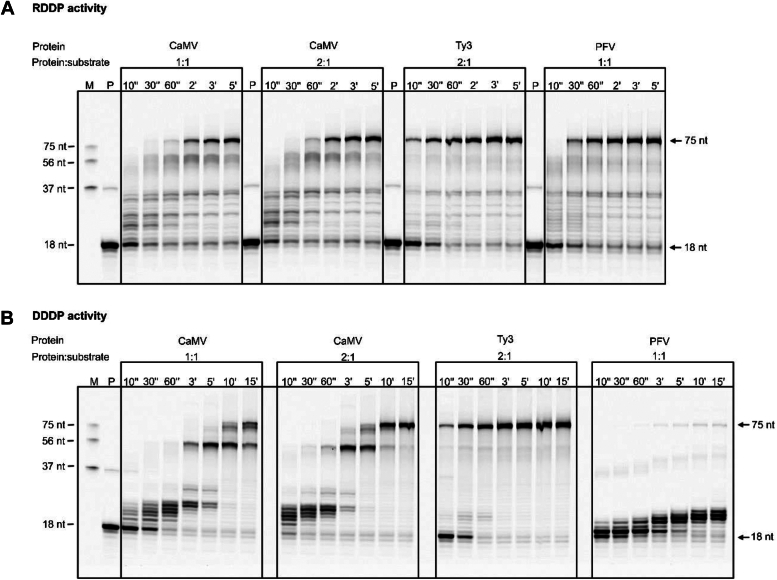
**Polymerase activity assay.***A*, RNA-dependent DNA polymerase (RDDP) activity assay. The CaMV RT polymerase activity assay was performed at different enzyme concentrations (100 and 200 nM) with 100 nM hybrid substrate (fluorescently 5ʹ labeled 18 nt DNA primer hybridized to the 3ʹ terminus of a 75 nt RNA template). The reaction was performed at different time intervals (10 s, 30 s, 60 s, 2 min, 3 min, and 5 min, marked on top of each lane) at 30 °C. Ty3 and PFV RT polymerase activity was determined at 2:1 and 1:1 protein:substrate molar ratios (200 nM:100 nM, 100 nM:100 nM, indicated on top of each time-course), respectively. The reaction products were analyzed on urea-PAGE gels with fluorescence detection. Lane M: DNA markers. Lane P: fluorescently labeled DNA primer. *B*, DNA-dependent DNA polymerase (DDDP) activity assay. The assay was performed as in (*A*) with dsDNA substrate (fluorescently 5ʹ labeled 18 nt DNA primer hybridized to the 3ʹ terminus of a 75 nt DNA template). The reaction was performed at different time intervals (30 s, 60 s, 3 min, 5 min, 10 min and 15 min, marked on top of each lane) at 30 °C.

We first tested DNA polymerization on an RNA template (RNA-dependent DNA polymerase activity [RDDP]). We set up reactions with different concentrations of enzyme and a fixed concentration of substrate (100:100 nM and 200:100 nM protein:substrate concentration). The results showed that 21% of the DNA primer was extended to full-length products within 2 min, and 50% of the substrates were converted to a full-length product after 3 min. The rate of reaction was very similar between reactions that were performed at different protein:substrate ratios (Fig. 5*A*). This indicates that a single molecule of CaMV RT can perform the full polymerase function under the present conditions.

We also performed a reaction for Ty3 RT at a 2:1 molar ratio of protein to the hybrid substrate (Ty3 RT interacts with RNA/DNA as a dimer). The DNA primer extended to the full-length product within 30 s, so the reaction was much faster than for CaMV RT, which may be a result of Ty3 RT homodimerization. PFV RT polymerase activity was determined at a 1:1 ratio of enzyme to substrate concentration (PFV RT interacts with RNA/DNA as a monomer). The DNA primer completely extended within 60 s, again faster than for CaMV RT. These results suggested that CaMV RT RDDP activity is slower than that of Ty3 and PFV RTs.

We then used a DNA template to test DNA-dependent DNA polymerase activity (DDDP). CaMV RT was again tested at two protein:substrate molar ratios (1:1 and 2:1). The results showed that 52% of the DNA primer was converted to intermediate products within 5 min, and 28% of the substrate was converted to a full-length product after 10 min. Again, there was no major difference in polymerase activity at the 100 and 200 nM enzyme concentrations (Fig. 5*B*). This indicates that a single molecule of CaMV RT can synthesize the full-length product efficiently. The result of the Ty3 RT polymerase activity assay showed that most of the DNA primer was converted to the full-length product within 60 s. PFV RT converted only 2% of the DNA primer to full-length product after 15 min at a 1:1 molar ratio of enzyme to substrate. These results indicated that CaMV RT DDDP activity is slower than Ty3 RT but faster than PFV RT.

To ensure that all primer strands are hybridized to the template, we used an excess of the latter. We still observed unprocessed primer for all three tested RTs. Similar effect has been observed before for HIV-1 RT and Ty3 RT (40, 41). We currently do not have an explanation for this phenomenon but it appears to be a common feature of RTs.

To learn whether CaMV RT is a processive enzyme, we performed polymerization assays in the presence of the heparin trap. We used the same substrates as described above for the polymerase activity tests. We first tested different heparin concentrations and 4 μg/ml was the highest concentration at which residual RDDP activity was observed. We tested two protein:substrate ratios—1:2 and 1:1. Both RDDP and DDDP activities were strongly affected by the addition of heparin. The results of the RDDP activity assay showed that in the presence of 4 μg/ml heparin and at 1:1 molar ratio of enzyme to substrate, the RT was unable to convert the primer to full-length products. However, when we increased the enzyme concentration to 200 nM (2:1 protein:substrate ratio), we observed a small amount of full-length products after 3 min (Fig. S8*A*). DDDP activity assay showed that at 1:1 and 2:1 protein:substrate ratio, the enzyme was unable to convert the primer to full-length products in the presence of 4 μg/ml heparin and most of the primer was not extended (Fig. S8*B*). These results suggest that CaMV RT has poor processivity. By contrast, HIV-1 RT was still active in the presence of 1 mg/ml heparin (42). This shows that CaMV RT polymerase activity is much more sensitive to the presence of a heparin trap.

### RNase H activity assays

**Figure 6:**
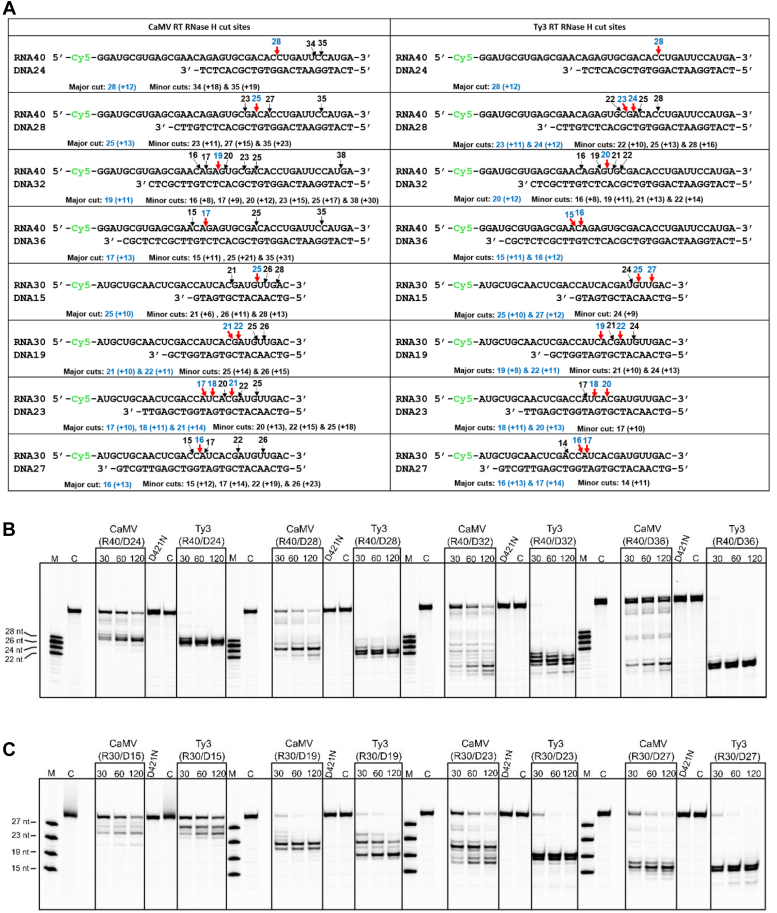
**RNase H activity assays.***A*, sequences of RNA/DNA substrates used in the experiments. Names of the substrates come from RNA (R) or DNA (D) and the length (in nt). In the *left* column, the cut sites for CaMV RT RNase H are marked with arrows (*red* arrows indicate major cuts with product lengths in nt given in *blue*; *black* arrows and labels show the sites of minor cuts). In the *right* column, the cut sites for Ty3 RT RNase H are shown. In both columns, the cuts are summarized below substrate sequence and the distance from the polymerase active site is given in parentheses. *B*, CaMV and Ty3 RTs RNase H activity assay for R40 series (R40/D24, R40/D28, R40/D32, and R40/D36) with 200 nM enzyme and 100 nM hybrid substrate. The reaction was performed at different time intervals (30, 60, and 120 min) at 30 °C. Reaction products were analyzed by urea-PAGE with fluorescence detection. Lane M: marker. Lane C: substrate with fluorescently labeled RNA. D421N: CaMV RH mutant control. The experiments were performed three times. *C*, CaMV and Ty3 RTs RNase H activity assay for R30 series (R30/D15, R30/D19, R30/D23, and R30/D27) performed as in (*B*).

Next, we biochemically characterized the RNase H activity of CaMV RT. We sought to verify whether the position of cuts that were introduced by CaMV RT RNase H is directed by the interaction of the recessed end of the DNA primer with the polymerase active site, which is a characteristic feature of many RTs (43). For these experiments, we used two RNAs of different lengths (30 and 40 nt) and sequences that were fluorescently labeled on the 5′ end. These RNAs were hybridized with four DNAs of different lengths that were complementary to the 3' portion of each RNA. RNA/DNA hybridization resulted in substrates in which one end was blunt and the other end contained 5′ RNA overhangs of different lengths (Fig. 6*A*). We reasoned that regardless of the length and sequence of the RNA/DNA, the positions of the RNase H cuts should be introduced at a fixed distance from the recessed 3' end of the DNA bound at the polymerase active site.

We first used substrates in which 40 nt RNA was hybridized to four DNAs (D24, D28, D32, and D36; Fig. 6*B*). For the R40/D24 and R40/D28 substrates, the major products of CaMV RNase H activity were 28 and 25 nt in length, respectively, and these products were generated by RNase H cuts 12 and 13 nt from the polymerase active site. For the R40/D32 substrate, the major product length was 19 nt and generated by an RNase H cut 11 nt from the polymerase active site. For the R40/D36 substrate, the major product length was 17 nt, which resulted from an RNase H cut 13 nt from the polymerase active site. The same set of substrates was also used to assay Ty3 RT RNase H activity. For the R40/D24, R40/D28, R40/D32, and R40/D36 substrates, the major product lengths were 28 (R40/D24), 23 and 24 (R40/D28), 20 (R40/D32), 15 and 16 nt (R40/D36), corresponding to products that were generated by RNase H cuts 11 and 12 nt from the polymerase active site.

The next experiment was conducted with 30 nt RNA hybridized to four different complementary DNAs (D15, D19, D23, and D27; Fig. 6*C*). For the R30/D15, R30/D19, R30/D23, and R30/D27 substrates, CaMV RT RNase H activity resulted in major products with the lengths of 25 (R30/D15), 21 and 22 (R30/D19), 17, 18 and 21 (R30/D23), and 16 nt (R30/D27) and these products were generated by cuts 10 to 14 nt from the polymerase active site. The Ty3 RT RNase H activity assay with the same substrates showed multiple major products that were generated by RNase H cuts 10 to 14 nt from the polymerase active site.

We next wished to monitor the RNase H activity during DNA polymerization to further confirm that it is the distance to the polymerase active site that is the most important factor determining the position of the RNase H cuts (44). We used four hybrid substrates from our previous experiment: R40/D24, R40/D28, R30/D15, and R30/D19, which contained a 5′-Cy5 label in the RNA strand and a 5′-Cy3 label in the DNA strand. This double-labeling approach allowed us to simultaneously monitor primer extension and template digestion. We added only three selected dNTPs to each substrate to limit the length of the primer extension. For example, R40/D24 was mixed with dCTP, dGTP, and dTTP to allow extension up to 8 nt (Fig. S9*A*). R40/D28 was extended by up to 4 nt, R30/15 by 4 nt, and R30/19 by 5 nt. When the RNase H cut sites were mapped, we observed that they moved closer to the 5′ end by a number of nucleotides similar to the number of nucleotides incorporated into the primer (Fig. S9, *B* and *C*). These results indicate that RNase H cuts are primarily determined by the interaction of the polymerase active site with the recessed 3ʹ end of the primer. However, the distance between the polymerase active site and the RNase H cuts is not strictly maintained during nucleotide addition by the polymerase domain. This indicates some flexibility in the positioning of the RNase H domain. Most likely, the sequence preference of the RNase H domain is an additional factor in the selection of the cut sites.

**Figure 7:**
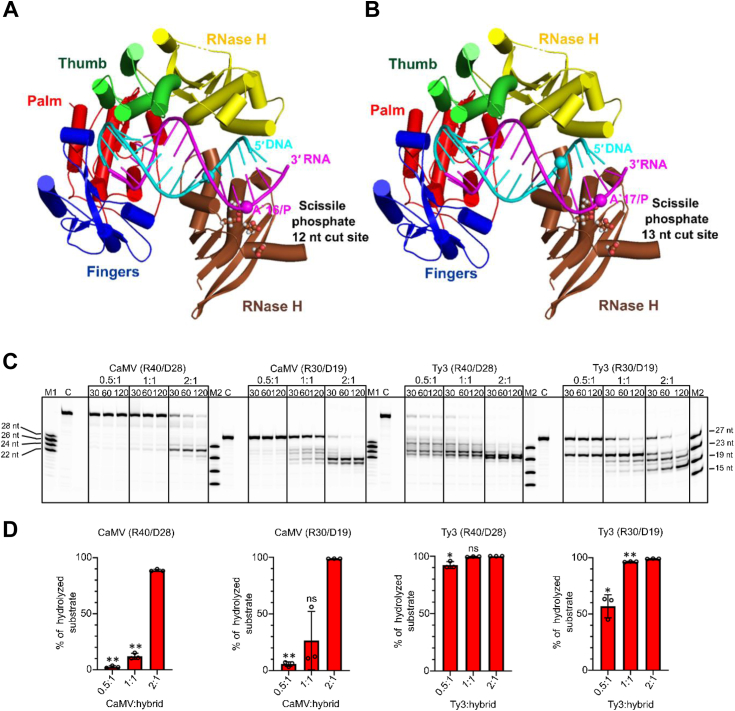
**CaMV RNase H functional models and activity assays.***A* and *B*, cartoon representation of CaMV RT RNA/DNA complex structure with an additional RNase H domain modeled to bind the hybrid with its active site positioned over nucleotide 16 (*A*) or 17 (*B*) of the RNA strand (cuts 12 and 13 nt from the polymerase active site, respectively). *C*, CaMV and Ty3 RTs RNase H activity assay performed at different protein:substrate ratios using R40/D28 and R30/D19 substrates. Enzymes at 50, 100, and 200 nM concentrations were mixed with 100 nM hybrid substrate, and the reaction was performed as in Figure 6, *B*–*D*, quantification of the data shown in (*C*). The amount of hydrolyzed substrate is plotted for the last time-point and different protein:substrate ratios. The error bar corresponds to the SD from three independent experiments and data point are shown as *circles*. Statistical comparisons between the 2:1 wt:hybrid RNase H activity and the activity of the other different ratios (0.5:1 wt:hybrid, 1:1 wt:hybrid) at the 120 min time point were performed using a paired sample *t* test. Significant differences (*p*-value < 0.05) are indicated by one asterisk (∗), highly significant differences (*p*-value < 0.01) by two asterisks (∗∗), and nonsignificant comparisons by "ns".

In Ty3 RT, the subunit B of the dimer provides RNase H activity. Compared to the available crystal structure, its RNase H domain must change its position to reach the scissile phosphate. In the structure of CaMV RT, the protein interacts with the substrate as a monomer with a configuration that is similar to chain A of Ty3 RT. Moreover, the active site of the RNase H domain in the crystal structure is disorganized, and we assume that this conformation is inactive. Therefore, we sought to determine the way in which CaMV RT RNase H activity can be executed. We first modeled a catalytic complex of the RNase H domain with an RNA/DNA hybrid. In our biochemical assays, we observed major cuts 12 and 13 nucleotides from the polymerase active site. We superimposed a structure of bacterial RNase H1 (PDB ID: 1ZBI) (45) on the RNA strand of the hybrid in our structure so that the active site would interact with the phosphate group 12 or 13 bp from the polymerase active site (Fig. 7, *A* and *B*). We then superimposed the CaMV RNase H domain on bacterial RNase H1. In this model, the CaMV RNase H can be neatly accommodated in the CaMV RT–RNA/DNA complex without any major steric clashes. This modeling exercise also clearly showed that the RNase H domain of CaMV RT in our structure cannot undergo a conformational change that would allow it to interact with the scissile phosphate. The linker between the polymerase and RNase H domain is too short to accommodate rearrangement of the RNase H domain to bring it to the scissile phosphate. Therefore, we hypothesized that another CaMV RT molecule provides RNase H activity by transiently interacting with the CaMV RT molecule that adopts a configuration that is observed in our structure.

To find the CaMV enzyme composition with hybrid substrate during RNase H hydrolysis, we performed RNase H cleavage assays using R40/D28 and R30/D19 substrates and different protein concentrations (50, 100, and 200 nM) while keeping the substrate concentration constant (100 nM; Fig. 7, *C* and *D*). For both tested substrates, the reaction was very slow for 1:2 and 1:1 protein:substrate molar ratios. In contrast, nearly complete degradation of the RNA occurred after 120 min of the reaction when a two-fold excess of protein was used. These results indicate that CaMV RT RNase H cleavage occurs efficiently only when excess protein over substrate is present. A very different result was obtained for Ty3 RT. For the R40/D28 substrate, the majority of the RNA was digested by the enzyme at a 50 nM concentration (1:2 protein:substrate molar ratio) after a 30 min reaction. For R30/D19 substrate, complete digestion was observed for the 100 nM protein concentration (1:1 RT:hybrid molar ratio) after 120 min. This shows that excess protein is not required for the efficient RNase H activity of Ty3 RT. We propose that this results from the fact that Ty3 RT forms a stable dimer when it interacts with RNA/DNA with one of the subunits carrying RNase H activity (6). In contrast, for CaMV RT, the protein preferentially and stably interacts with the substrate in the polymerase-competent but RNase H–inactive mode. To execute RNase H activity, an additional molecule from the excess pool of the protein is required. Indeed, the results of the RNase H activity assay showed that only in the presence of a molar excess of protein, the RNA/DNA substrate can be hydrolyzed efficiently by CaMV RT.

To provide further support for transient dimer formation, we extended the studies of RNase H cleavage at different protein:substrate ratios by including a reaction in which the substrate was mixed with WT protein and the inactive RNase H variant at equimolar concentrations. We reasoned that in such a wt/mutant mixture, four configurations of the transient dimer are possible: (i) homodimer of wt enzyme: wt molecule binds substrate in polymerase competent configuration and wt molecule transiently associates to perform the RNase H cuts, (ii) heterodimer of wt and mutant RTs: wt enzyme adopts the polymerase configuration and mutant enzyme molecule is transiently associated, (iii) heterodimer of wt and mutant RTs: mutant enzyme molecule adopts the polymerase configuration and wt enzyme molecule transiently associates, (iv) a homodimer of two mutant enzyme molecules is formed (Fig. S10*A*). Only half of the possible configurations will include a transiently associated wt enzyme that could provide the RNase H activity. This would reduce the efficiency of RNase H cleavage by wt/mutant mixture by half compared to wt enzyme mixed with the substrate at 2:1 molar ratio.

To test this, we used R40/D28 substrate. The observed results were in agreement with our prediction: there was almost complete RNA cleavage of after 120 min of reaction when wt protein was used at 2:1 protein:substrate molar ratio. When a 1:1:1 mixture of wt RT:mutant RT:substrate was used, the reaction efficiency was reduced to ∼60%. As expected, no cleavage was observed when the mutant protein was mixed with the substrates at a 2:1 ratio. As shown in the previous RNase H activity assays, when we used 1:1 wt enzyme:substrate, only ∼18% of the activity was observed (Fig. S10, *B* and *C*). We reasoned that in this case, most of the enzyme is sequestered by stable association in the polymerase configuration and no protein subunits are available for transient association and RNase H cleavage. This is an important observation. In fact, a 1:1:1 reaction (wt/mut) contains the same amount of both the wt RT and substrate as the standard 1:1 reaction and yet, we see a much higher cleavage rate (∼60% *versus* ∼18%). This means that mutant enzyme can fulfill a function which facilitates RNase H activity. These results further support our hypothesis that one RT molecule binds the substrate in the polymerase configuration and transiently positions another RT molecule, which is required for RNA hydrolysis.

**Figure 8:**
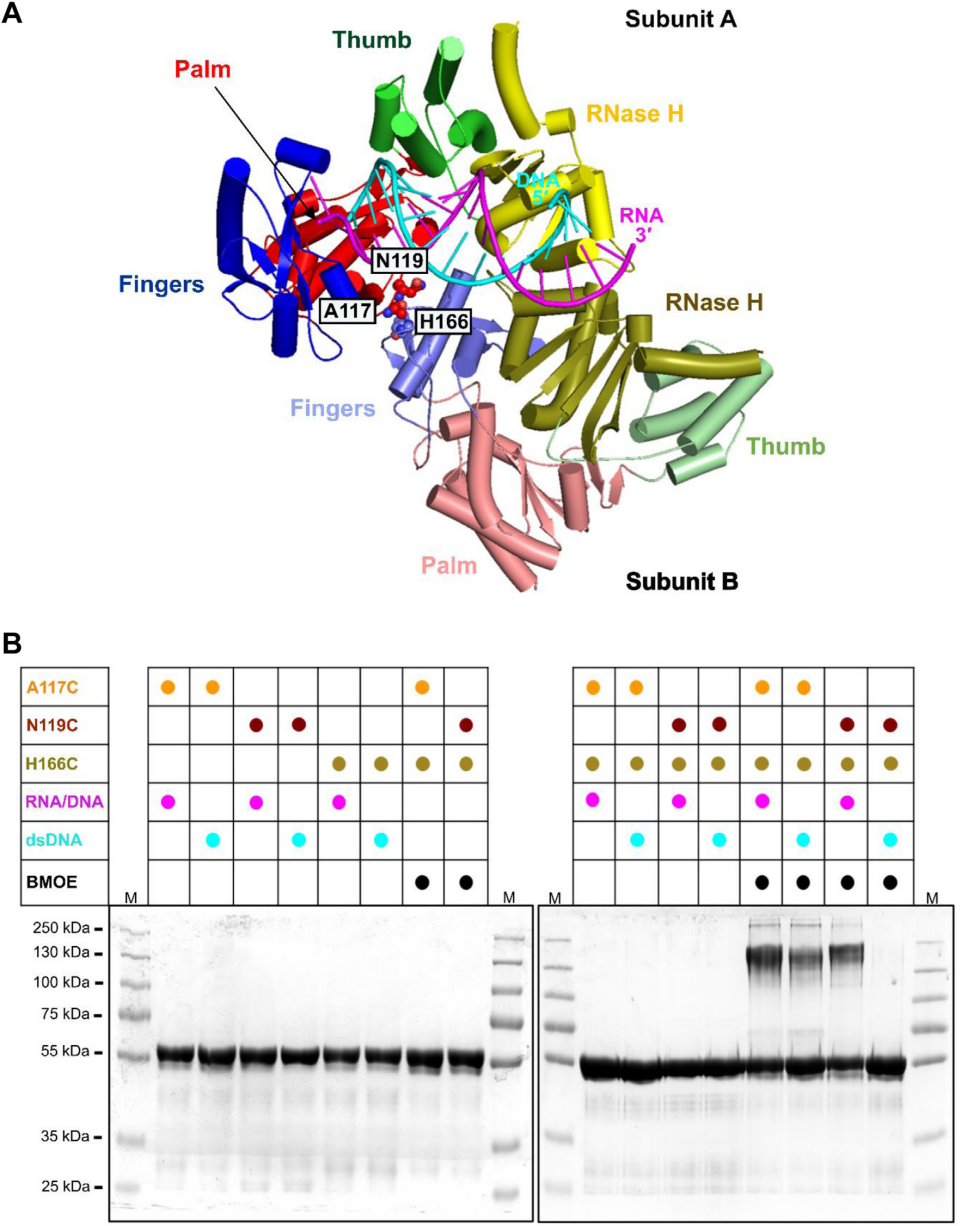
**Cysteine-based chemical cross-linking of CaMV RT.***A*, cartoon representation of a model of CaMV RT dimer in complex with RNA/DNA generated by AlphaFold 3. Subunit A is colored as in Figure 1*A* and subunit B is shown in lighter shades of the same colors. Residues substituted with cysteines are shown as *spheres* and labeled. *B*, chemical cross-linking reactions with A117C, N119C, and H166C variants of CaMV RT. The components of each reaction are indicated on *top* of the gel. 2:1 protein:substrate molar ratio was used and 4.5 μM bismaleimidoethane (BMOE) was included in the indicated reactions. All reactions were incubated at room temperature for 120 min and the products were analyzed by 9% SDS-PAGE.

We propose that the RT molecule that executes RNA hydrolysis is positioned through interaction with the RT molecule that binds the substrate in the polymerase configuration (*i.e.*, the complex in our structure). Our biochemical experiments show that RNase H cleavage by CaMV RT occurs 10 to 14 nt from the polymerase active site. This distance is similar to that observed for Ty3 RT. Therefore, we can assume that the configuration of the transient CaMV RT is similar to the dimer structure of Ty3 RT, which is corroborated by modeling using AlphaFold 3 (36) (Fig. 8*A*). However, we note that in the Ty3 RT structure (PDB ID: 4OL8), RNase H must undergo a conformational change to reach the backbone of the RNA. Therefore, the structure of the conformation conducive to RNase H cleavage has not been determined for either CaMV RT or Ty3 RT, and further structural studies are needed to provide this information.

To further demonstrate CaMV RT dimerization, we designed a cysteine-based chemical cross-linking approach using the AlphaFold 3 model of the CaMV RT dimer interacting with RNA/DNA (Fig. 8*A*). In the CaMV RT model, we identified potential contacts between the subunits of the dimer. For the subunit in the polymerase configuration (configuration in our structure), the contact was mediated by residues Ala117 and Asn119. In the transiently associated subunit, which contributes RNase H activity, the contact was mediated by residue His166. All three residues were individually substituted with cysteines. We then mixed the H166C variant with A117C or N119C in the presence of a bifunctional thiol cross-linker, bismaleimidoethane, and RNA/DNA or dsDNA. Both combinations of variants yielded cross-linked products in the presence of the RNA/DNA hybrid substrate (Fig. 8*B*). However, for the reaction with dsDNA, the cysteine mutants of the A117C/H166C combination produced a smaller amount of the cross-linked product, while the N119C/H166C combination did not form cross-linked products. This shows that for CaMV RT, the RNA/DNA hybrid is more efficient in promoting dimer formation. This is consistent with our hypothesis that the function of dimer formation is to bring RNase H into position for RNA cleavage.

In conclusion, CaMV RT RNase H activity shows polymerase-directed cut selection. This activity results from the transient association of a second CaMV RT whose RNase H domain performs RNA hydrolysis.

## Discussion

We determined the structure of CaMV RT in complex with an RNA/DNA hybrid. It is the first crystal structure of an RT from a plant-infecting dsDNA virus of the pararetrovirus family. The overall structure of the enzyme is very similar to Ty3 RT. CaMV RT interacts with a hybrid substrate at a 1:1 ratio, in contrast to the Ty3 RT that binds RNA/DNA hybrids as a dimer.

An interesting feature of our structure is the displacement of the last helix of the RNase H domain (helix M) from its expected position which leads to the disorganization of the RNase H active site. To verify the importance of the observed positioning of helix M, we prepared mutants of CaMV RT in which the thumb subdomain residues N330 and Y323 interacting with helix M were substituted with tryptophan and alanine, respectively. We worked with two CaMV RT variants: N330W and Y323A/N330W. Their RNase H activity was severely decreased (not shown). We currently cannot explain this observation. Further structural studies will be required to fully understand the role of helix M-thumb subdomain contacts.

The results of the RNase H assays showed that regardless of the length and sequence of the hybrid region, predominant cuts by the CaMV RT RNase H domain are located 10 to 14 nt from the polymerase active site. Similar results were obtained in the absence and presence of DNA polymerization. The fact that a range of cut sites was observed indicates the flexibility of CaMV RT. Alternatively, this can also result from two possible configurations of the substrate at the polymerase active site: pretranslation and post-translocation. In addition, the cut sites can be determined by sequence preference of the RNase H domain. These differences notwithstanding, the major cuts for various substrates occur at a constant distance range from the polymerase active site. The results for Ty3, in agreement with previous data (6, 46), also showed that RNase H cuts are determined by the distance from the polymerase active site. The distance range was similar to CaMV RT and was 10 to 14 nt, but the rate of reaction was different between these two enzymes.

The results of the RNase H activity assays with different molar ratios of enzyme and substrate (0.5:1, 1:1, and 2:1) suggest that two enzyme molecules are required for the CaMV RT RNase H domain to be functional. One molecule is thus involved in substrate binding, and another is involved in hydrolysis. Most of the observed CaMV RT RNase H cuts are directed by the interaction of the recessed 3′ end of the bound DNA with the polymerase active site and do not occur throughout the RNA/DNA. This indicates that the RT molecule that executes RNA hydrolysis is positioned through interaction with the RT molecule that binds the substrate in the polymerase configuration (*i.e.*, the complex in our structure). However, the association of this second, RNase H-competent CaMV RT molecule must be transient and thus could not be captured in the crystal structure or detected in gel filtration or AUC experiments.

CaMV RT and Ty3 RT operate through different mechanisms. CaMV RT preferentially and stably associates with a hybrid substrate in monomeric form and in polymerase configuration, so for a 1:1 molar protein:substrate ratio at the beginning of the reaction, the vast majority of protein molecules are in the polymerase configuration and no/very little excess enzyme is left to perform the RNase H function. At a 2:1 protein:substrate ratio, the excess protein will be able to weakly/transiently associate with CaMV RT, which interacts with the hybrid in the polymerase configuration, and cleave the RNA. For Ty3 RT, the preferred organization is a substrate-induced dimer. We predict that at the beginning of the reaction for a 2:1 protein:substrate ratio, almost all hybrids will be bound and cleaved by Ty3 RT dimers, for a 1:1 ratio, about half of the hybrids will be bound and cleaved by Ty3 RT dimers, and finally for a 1:2 protein:substrate ratio, about 25% of the hybrids will be bound and cleaved by the Ty3 RT dimers. These predictions agree with our observation that the highest Ty3 RNase H activity is observed at 2:1 ratio but at 1:1 and 1:2 ratios, RNase H cuts are still observed. This is in contrast to CaMV RNase H, which is only active when excess protein is present.

A key element of the RT mechanism is the fine-tuning of RNase H activity. The nuclease cannot be engaged at the substrate constitutively because this would affect the processivity of DNA polymerization. Various mechanisms have been described to restrict the access of RNase H to the RNA/DNA duplex. For HIV-1 RT, a conformational change of the substrate is required to bring it into the RNase H active site (47). In MFV and XMRV RTs, RNase H is linked to the connection domain with a long flexible linker. This RNase H is mobile and only occasionally interacts with the substrate (12, 14). For Ty3, a substantial conformational change of subunit B is required to bring RNase H to the substrate. The CaMV RT mechanism that is described herein presents another way of RNase H regulation. Our data suggest a model in which a second copy of the enzyme transiently interacts with the RT molecule that binds the substrate in the polymerase configuration. The exact conformation of the second RT molecule is unknown. However, available structural data show a large degree of flexibility of RTs, so this conformation may be very different from the one that was observed in our structure and may be similar to the one of subunit B of Ty3 RT.

RTs that have been described to date exhibit very different oligomerization states and can work either as monomers or constitutive or substrate-induced dimers. For foamy viral MFV RT, the oligomerization state depends on the nucleic acid that is bound (*i.e.*, the enzyme is monomeric on RNA/DNA and dimeric on dsDNA). These differences affect the RNase H domain mechanism but can also change properties of the polymerase (*e.g.*, affecting its processivity). It would be interesting to decipher factors that determine the oligomeric state of various RTs. One factor can be the structure of the RNase H domain. Retroelement and caulimoviral RTs comprise an ancient RNase H domain. In retroviral RT, a new RNase H domain was acquired. This new RNase H in monomeric RT comprises a characteristic nucleic acid–binding element, termed basic protrusion, whereas some dimeric RTs possess an RNase that lacks it. Other factors that determine the oligomerization status can be the replication cycle of the virus and whether its genetic material is encoded in ssRNA (reverse transcription occurs early in the cycle) or dsDNA (reverse transcription occurs late). Systematic biochemical studies of various RTs will be required to elucidate oligomerization state determinants for these enzymes.

In summary, we provide the first structural and biochemical information for caulimoviral RT. These viruses are unique because they possess a dsDNA genome and use an ancient, small form of an RT that has the same composition as the LTR retroelement enzyme. Our data show that the structure of CaMV RT is very similar to Ty3 enzyme, but it functions as a monomer during DNA polymerization. RNase H activity requires the transient association of another copy of CaMV RT, which is a new mode of RNase H activity regulation.

## Experimental procedures

### Cloning, protein production, and purification

The CaMV RT polypeptide chain length is 479 amino acids. A synthetic gene encoding CaMV RT with an N-terminal 6XHis-SUMO tag cloned into the pET28 expression vector was purchased from GenScript. The expression constructs for CaMV RT variants: RNase H mutant D421N and variants for chemical cross-linking (A117C, N119C, and H166C) were generated using site-directed mutagenesis PCR to introduce amino acid changes. The CaMV RT proteins (wt and point substitution variants) were expressed in *Escherichia coli* BL21 (RIL) overnight at 16 °C after induction with 0.3 mM IPTG. Cells were harvested by centrifugation at 3985 relative centrifugal force (rcf) for 20 min at 4 °C and then resuspended in buffer A that consisted of 50 mM Hepes (pH 7.5), 0.5 M NaCl, 20 mM imidazole, 5 mM β-mercaptoethanol, and 10% glycerol (w/v). A protease inhibitor mixture was added along with 1 mg/ml lysozyme enzyme, and the suspension was incubated on ice for 30 min. The cells were lysed by sonication, and insoluble debris and membranes were removed by centrifugation at 72,530 rcf for 40 min. Supernatant was loaded on 5 ml Ni-NTA column (HiTrap, GE Healthcare) equilibrated with buffer A. The column was washed with 10 column volumes of buffer A. His-tagged CaMV RT protein was eluted from the column using a step gradient of imidazole from 20 to 500 mM in the increments of 100 mM per 5 ml. Protein purity was analyzed by SDS-PAGE on a 9% acrylamide gel. To remove the 6xHis-SUMO tag, the fusion protein was digested with SUMO protease (Ulp1) and dialyzed overnight against buffer A at 4 °C. The cleaved-off His-tagged SUMO protein was removed on a His-Trap column (GE Healthcare). The tag-free protein from the flow-through was concentrated using a centrifugal filter device with a 10 kDa cut-off (Millipore) and applied to a Hiload 16/600 Superdex 200 size-exclusion column (GE Healthcare), pre-equilibrated with buffer A without imidazole. The pure protein fractions were combined and concentrated using a Millipore 10 kDa cut-off centrifugal filter device to a final concentration of 10 mg/ml. The protein was then stored at −80 °C for further studies. Protein concentration was determined by measuring the absorbance at a wavelength of 280 nm.

### Crystallization, data collection, and structural solution

Initial crystallization studies for CaMV RT were conducted using the sitting-drop vapor diffusion method at 18 °C. Crystallizations were performed for the protein alone (D421N variant) and in the presence of RNA/DNA hybrids, ranging from 12 to 24 base pair (bp) in length. All oligonucleotides were purchased from Eurofins GATC. Before crystallization, the protein and RNA/DNA hybrids were mixed in a 1:1 molar ratio at a final protein concentration of 4 mg/ml. Protein was diluted with a buffer that contained 20 mM Hepes (pH 7.5), 200 mM NaCl, 5% glycerol, and 5 mM MgCl_2_. The first crystals were grown in a JCSG Plus screen (Jena Bioscience) for the complex containing a hybrid formed by RNA R18 (5′-GGUCCAGCAGUGCGUAGC) and DNA D16 (5′-GCTACGCACTGCTGGA) oligonucleotides. The best diffracting crystals were obtained by the hanging-drop vapor diffusion method in a buffer condition of 0.04 M citric acid, 0.06 M BIS-Tris propane (pH 5.5), and 20% (w/v) PEG 3350.

The X-ray diffraction data were collected at the PETRA III storage ring at the P11 beamline (48, 49). Data were processed and scaled by XDSAPP GUI (50). To solve the complex structure, we used the PHASER molecular replacement method (51), which is part of the PHENIX software suite (52). The apo-CaMV RT model structure that was generated by AlphaFold (35) was used as a search model.

Superposition of CaMV RT AlphaFold model and crystal structure resulted in an RMSD of 4.1 Å over 470 Cα atoms. Superposition of individual domains of the CaMV RT AlphaFold model and the final refined crystal structure showed high similarity in the polymerase domain (RMSD of 1.0 Å for 258 pairs of Cα atoms) as well as in the thumb (RMSD of 2.2 Å for 75 pairs of Cα atoms) and RNase H (RMSD of 2.4 Å for 128 pairs of Cα atoms) domains. One difference is the position of the polymerase primer grip consisting of the β-hairpin domain, which in our structure is slightly shifted towards the active site of the polymerase due to interactions with the primer strand. The last α-helix of the RNase H domain (the M-helix) is removed from its canonical position in the domain fold in both the AlphaFold model and in the structure but is located slightly closer to the thumb domain in the crystal structure.

### Structural and bioinformatics analysis

Structural analyses were performed using the PyMol (The PyMOL Molecular Graphics System, version 4.6.0, X.Y Schrödinger) and University of California, San Francisco, Chimera (53) visualization tools. Electrostatic surface analyses were performed using the Adaptive Poisson-Boltzman solver and calculated using the APBS plugin for PyMol (https://pymolwiki.org/index.php/APBS). Multiple and pairwise sequence alignments were generated using the Clustal omega tool (https://www.ebi.ac.uk/Tools/msa/clustalo/). Structure-guided multiple sequence alignments were generated with PROMALS3D (54).

### Fast protein liquid chromatography analysis

For gel filtration chromatography studies of CaMV RT with the analyses of complex formation, we used the hybrid of RNA-24 (5′-GGCAGUGGUCCAGCAGUGCGUAGC) and DNA-22 (5′-GCTACGCACTGCTGGACCACTG) and a dsDNA substrate that was prepared using DNA-24 (5′-GGCAGTGGTCCAGCAGTGCGTAGC) and DNA-22. CaMV RT was mixed with the substrates at different molar ratios (0.5:1, 1:1, and 2:1) in a buffer that contained 25 mM Hepes (pH 7.5), 200 mM NaCl, 5% glycerol, 5 mM MgCl_2_, and 1 mM DTT. The mixtures were incubated on ice for 30 min and then applied to a Superdex 200 (10/300) gel filtration chromatography column to examine the formation of protein–nucleic acid complexes.

### Analytical centrifugation: Sedimentation equilibrium

For analytical centrifugation studies, we used the same hybrid and dsDNA substrates as in the gel filtration analysis. CaMV RT was mixed with substrates at a 1:1 molar ratio (2 μM:2 μM) in a buffer that contained 25 mM Hepes (pH 7.5), 200 mM NaCl, 5% glycerol, 5 mM MgCl_2_, and 1 mM DTT. The mixtures were incubated on ice for 30 min and then subjected to sedimentation equilibrium experiments to examine the formation of protein–nucleic acid complexes.

A Beckman-Coulter Optima ProteomeLab XL-I analytical ultracentrifuge that was equipped with an eight-place AN-Ti 50 rotor was used for sedimentation equilibrium experiments. A 12 mm cell with an Epon double sector centerpiece and sapphire windows was used, filled with 160 μl of the protein, hybrid, dsDNA, or complex samples and 170 μl of buffer. Samples were equilibrated at 20 °C at 8050 rcf, and equilibrium was monitored by taking scans every 4 h using the “test approach to equilibrium” procedure from the SEDFIT program (version 16.1c) (55). Data were acquired every 0.001 cm with 20 replicates using step scan mode. Scans were analyzed using the “species analysis” procedure of the SEDPHAT program (version 15.2 b). The partial specific volume (v-bar) of the protein (0.7429 ml/g) was calculated using the SEDNTERP program (56). In the case of hybrid and dsDNA, the literature value of 0.530 or 0.540 ml/g was used, or the v-bar of the protein (0.7429 ml/g) was used to calculate the buoyant molar mass.

The density (ρ = 1.023213 g/cm^3^) and viscosity (η = 1.201 mPa s) of the buffer were measured using an Anton Paar DMA 5000 densitometer (Graz, Austria) and an Anton Paar Lovis 2000 M/ME viscometer, respectively. The results were plotted using the GUSSI program (Chad Brautigam, UT Southwestern).

### RNase H assays

RNA and DNA oligonucleotides were purchased from Eurofins. Two groups of substrates were used for the activity assay. In the first group, fluorescently labeled RNA-40 (5′-Cy5-GGAUGCGUGAGCGAACAGAGUGCGACACCUGAUUCCAUGA) was hybridized with different lengths of complementary DNA (DNA-24: 5'- TCATGGAATCAGGTGTCGCACTCT; DNA-28: 5′-TCATGGAATCAGGTGTCGCACTCTGTTC; DNA-32: 5′-TCATGGAATCAGGTGTCGCACTCTGTTCGCTC; or DNA-36: 5′-TCATGGAATCAGGTGTCGCACTCTGTTCGCTCACGC). In the second group, fluorescently labeled RNA-30 (5′-Cy5-AUGCUGCAACUCGACCAUCACGAUGUUGAC) was hybridized with different lengths of complementary DNA substrates (DNA-15: 5′-GTCAACATCGTGATG; DNA-19: 5′-GTCAACATCGTGATGGTCG; DNA-23: 5′-GTCAACATCGTGATGGTCGAGTT; and DNA-27: 5′-GTCAACATCGTGATGGTCGAGTTGCAG). RNAs and DNAs were hybridized in H_2_O by heating to 85 °C for 3 min, followed by cooling to room temperature overnight. The hydrolysis reaction was initiated by adding the enzyme to a final concentration of 200 nM in a reaction buffer that contained 25 mM Tris–HCl (pH 7.8), 100 mM KCl, 10% glycerol, 9 mM MgCl_2_, and 1 mM DTT. The reaction mixture was then incubated at 30 °C for various time intervals (30, 60, and 120 min). The reaction was stopped by adding an equal volume of formamide with orange G loading dye and heated to 95 °C for 5 min. The hydrolyzed products were separated on denaturing PAGE (20% urea TBE-urea gel) and visualized on Amersham Typhoon Biomolecular Imager with Image Quant Total Lab software (GE Healthcare life sciences ImageQuant TL 1D v8.2.0, https://www.cytivalifesciences.com/en/us/shop/protein-analysis/molecular-imaging-for-proteins/imaging-software/imagequant-tl-10-2-analysis-software-p-28619). Fluorescently labeled RNA markers of different lengths (R40: 22, 24, 26 nt, and 28; R30: 15, 19, 23, and 27 nt) were used to measure the length of the products. All reactions were performed in triplicate.

To find the oligomeric state of the enzyme during RNase H cleavage, we performed experiments with two different lengths of hybrid substrates (R40/D28 and R30/D19). The hydrolysis process was initiated by adding the enzyme at three different concentrations (50, 100, and 200 nM) to 100 nM hybrid substrate in a reaction buffer that contained 25 mM Tris–HCl (pH 7.8), 100 mM KCl, 10% glycerol, 9 mM MgCl_2_, and 1 mM DTT. The reaction was performed at different time intervals (30, 60, and 120 min) at 30 °C. The hydrolyzed products were separated by denaturing PAGE (20% urea TBE-urea gel) and visualized on an Amersham Typhoon Biomolecular Imager with Image Quant Total Lab software (GE Healthcare). All reactions were performed in triplicate.

A variant of the RNase H activity assay was also performed to study this activity in the presence of DNA polymerization. The assay was performed as described above using four hybrid substrates: R40/D24, R40/D28, R30/D15, and R30/D19, which contained a 5′-Cy5 label in the RNA strand and a 5′-Cy3 label in the DNA strand. We added only three selected dNTPs to each substrate to limit the length of the primer extension. dCTP, dGTP, and dTTP (at a final concentration of 50 μM each) were used for R40/D24 (primer extension of 4 nt), R40/D28 (primer extension of 8 nt), and R30/D15 (primer extension of 4 nt). dATP, dGTP, and dTTP were used for R30/D19 (primer extension of 5 nt).

### Polymerase activity assay

The substrate for the polymerase activity assay was obtained by annealing the DNA-18 (5′-Cy5-AATAAACACCACGTGTGA) primer to RNA-75 (5′-GUCAGUGUGUUAAUCUUACAACCAGAACUCAAUUACCCCCUGCAUACACUAAUUCUUUCACACGUGGUGUUUAUU) or DNA-75 (5′-GTCAGTGTGTTAATCTTACAACCAGAACTCAATTACCCCCTGCATACACTAATTCTTTCACACGTGGTGTTTATT) template strands. We used a 20% excess of the template (1.2:1 template:primer molar ratio) to ensure that all the primer molecules were hybridized with the template. The reaction mixture contained 100 nM hybrid substrate, 0.2 mM dNTPs, 20 mM Tris (pH 7.8), 150 mM NaCl, 9 mM MgCl_2_, 5 mM DTT, and 10% glycerol. Polymerization was initiated by adding the enzyme to a final concentration of 100 or 200 nM and continued at 30 °C for the indicated times. The reaction was stopped by adding an equal volume of formamide with orange G loading dye and heated to 95 °C for 5 min. The polymerization products were separated by denaturing PAGE (20% urea TBE-urea gel) and visualized on an Amersham Typhoon Biomolecular Imager with Image Quant Total Lab software (GE Healthcare). Fluorescently labeled DNAs of different lengths (35, 55, and 75 nt) were used as markers to measure the length of the products. All reactions were performed in triplicate.

A variant of the above reaction was also performed in the presence of 4 μg/ml heparin trap which was added after mixing of the CaMV RT and nucleic acid and before the addition of MgCl_2_ and dNTP mix.

### Chemical cross-linking

For the cysteine-based chemical cross-linking experiment, we used the same hybrid and dsDNA substrates as used for FPLC/gel filtration analysis. The CaMV RT subunit variants A117C or N119C were mixed with the RNA/DNA hybrid or dsDNA at a 1:1 ratio (4.5 μM: 4.5 μM) and incubated on ice for 15 min, followed by the addition of the H166C variant (4.5 μM) and incubation on ice for 30 min. The cross-linking reaction was initiated by adding 4.5 μM bismaleimidoethane in reaction buffer containing 25 mM Tris (pH 7.0), 150 mM NaCl, 10% glycerol, 5 mM CaCl_2_, and 2.5 μM DTT. The reaction mixture was incubated at room temperature for 2 h. The reaction was stopped by adding DTT to the final concentration of 25 mM and incubating at room temperature for 15 min. The cross linked samples were separated in 9% SDS PAGE.

## Data availability

Atomic coordinates and structure factors for the reported CaMV RT crystal structure have been deposited in the PDB (ID: 8R0S).

## Supporting information

This article contains supporting information (6, 8, 11, 14, 35, 37, 45, 54).

## Conflict of interest

The authors declare that they have no conflicts of interest with the contents of this article.
