# Supplementary material for: Structural and biochemical characterization of cauliflower mosaic virus reverse transcriptase
Source: J Biol Chem. 2024 Jul 11;300(8):107555. doi: 10.1016/j.jbc.2024.107555 (PMC11363490; doi:10.1016/j.jbc.2024.107555)
Supplement: Supporting Information [file mmc1.pdf]

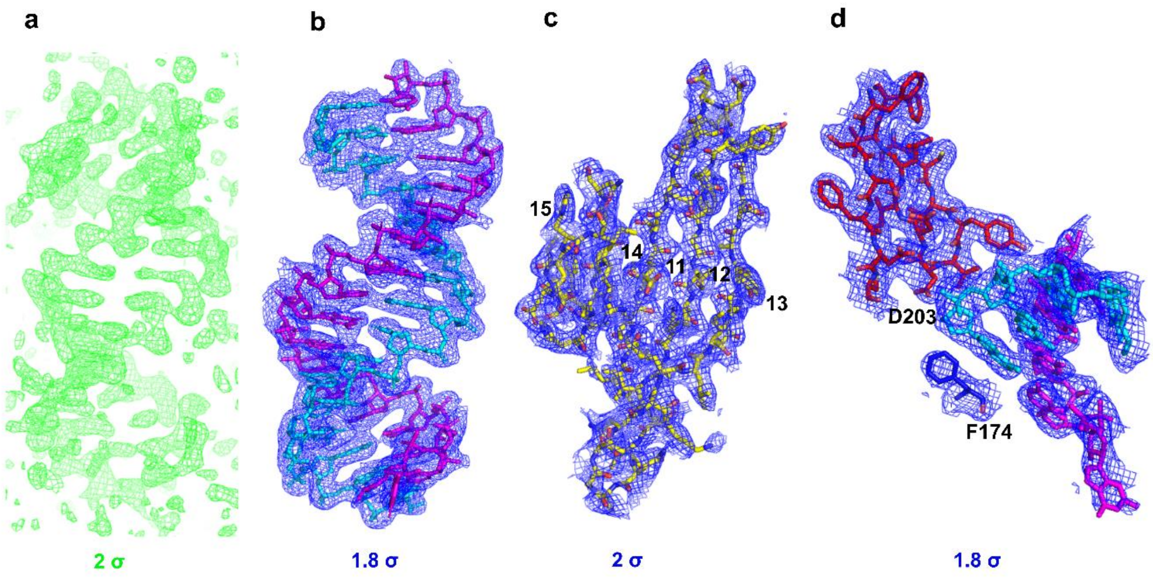

Supplementary Figure 1. Samples of electron density maps. (**a**) Fo-Fc map for the RNA/DNA hybrid after solving the structure using the molecular replacement method. AlphaFold 2 model of CaMV RT was used as the search model (1). (**b-d**) Samples of 2Fo-Fc simulated annealing composite omit electron density map corresponding to the RNA/DNA hybrid (**b**), central β-sheet of the RNase H domain (**c**) and the region of the structure around the DNA polymerase active site (**d**). Contour levels are given below the panels.

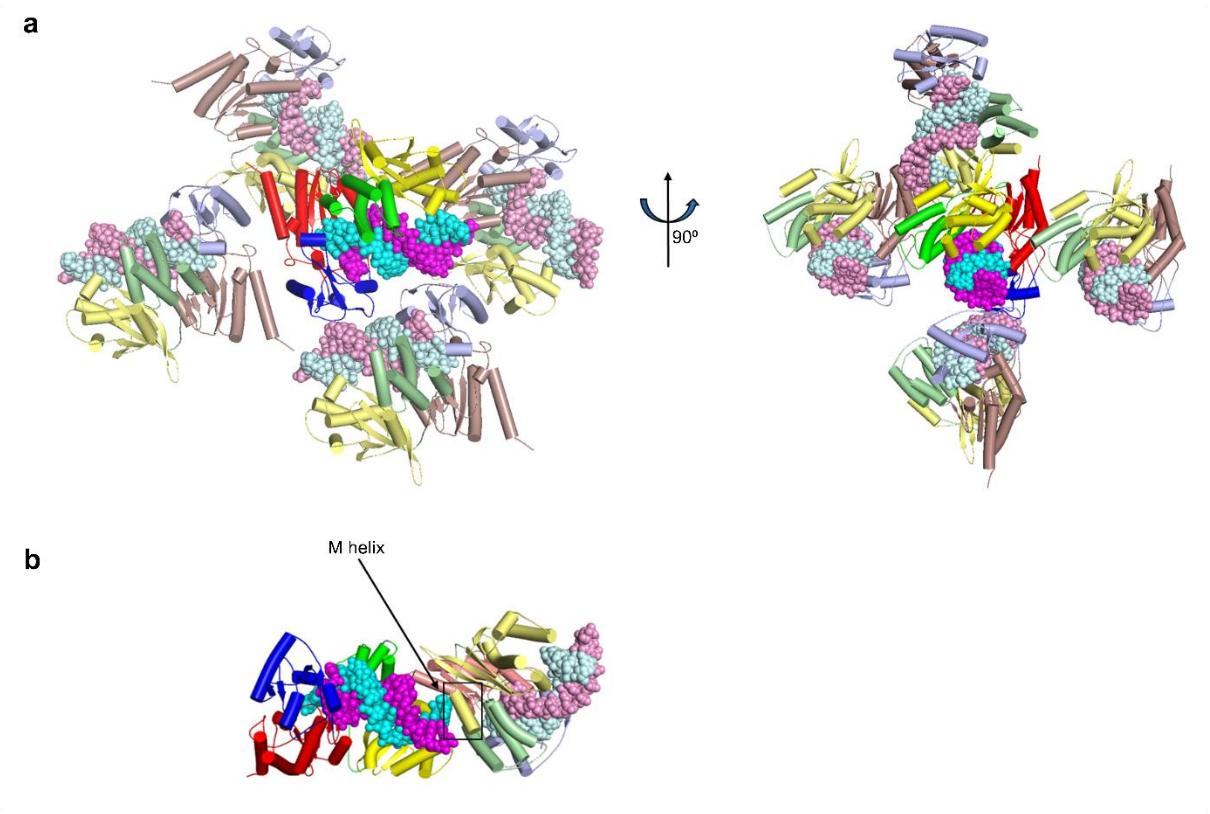

Supplementary Figure 2. Crystal packing of CaMV RT-RNA/DNA complex crystal. (a) Two views of the crystal packing. Only a single layer of molecules is shown in each view for clarity. The structure is colored as in Figure 1, with the protein shown as a cartoon and the RNA/DNA shown as spheres. Symmetry-related molecules are shown in lighter colors. (**b**) Close-up view of the RNA/DNA-helix M contact.

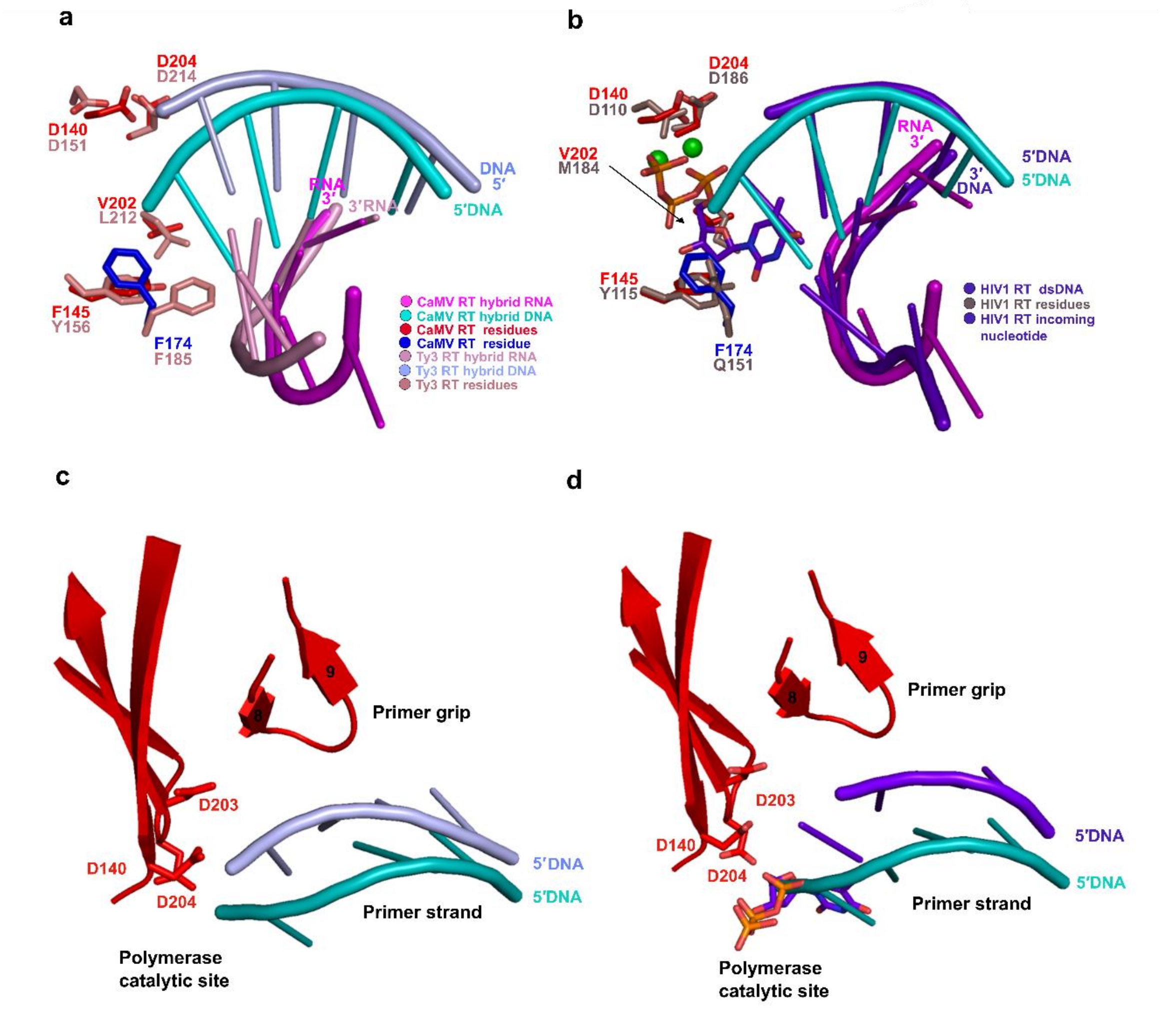

Supplementary Figure 3. Superposition of DNA polymerase active sites of different RTs. (**a**) Superposition of the polymerase active sites of CaMV RT and Ty3 RT. CaMV RT residues are shown in red and blue. Ty3 RT residues are shown in dark salmon (PDB ID: 4OL8) (2). RNA/DNA from the CaMV RT structure is shown as magenta (RNA) and teal (DNA) ladders, and the hybrid from the Ty3 RT structure is shown as pink (RNA) and light blue (DNA) ladders. (**b**) Superposition of the polymerase active site of CaMV RT and HIV-1 RT. HIV-1 RT residues are shown in dark salmon (PDB ID: 1RTD) (3). Two Mg^{2+} ions from the HIV-1 RT structure are shown as green spheres. The dsDNA from the HIV-1 RT complex structure is shown as a slate ladder. The incoming nucleotide from the HIV-1 RT structure is shown as slate sticks. (**c**) Superposition as in (a) but showing the difference in primer trajectory. Selected elements from

CaMV palm subdomain are shown as red cartoon. (**d**) Superposition as in (b) but showing the difference in primer trajectory. Selected elements from CaMV palm subdomain are shown as red cartoon.

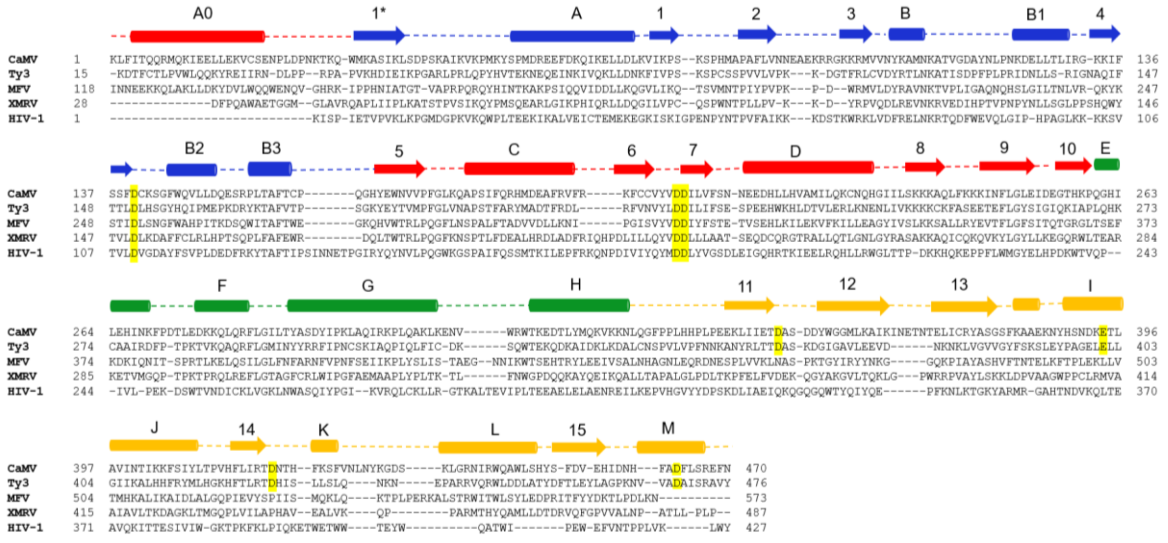

Supplementary Figure 4. **Structure-based multiple sequence alignment of selected RTs**. Alignment was generated with PROMALS3D (4) using the structures of RTs of CaMV, Ty3 (PDB ID: 4OL8) (2), HIV-1 (PDB ID: 1RTD) (3), XMRV (PDB ID: 4HKQ) (5), and MFV (PDB ID: 7O0G) (6). Secondary structure was calculated by PROMALS3D (4). For all sequences, active site residues are highlighted in yellow. Secondary structure elements are shown as cylinders (α-helices) and arrows (β-strands) and labeled and colored as in Figure 1 in the main text. For retroviral RTs, the RNase H domain sequence is omitted.

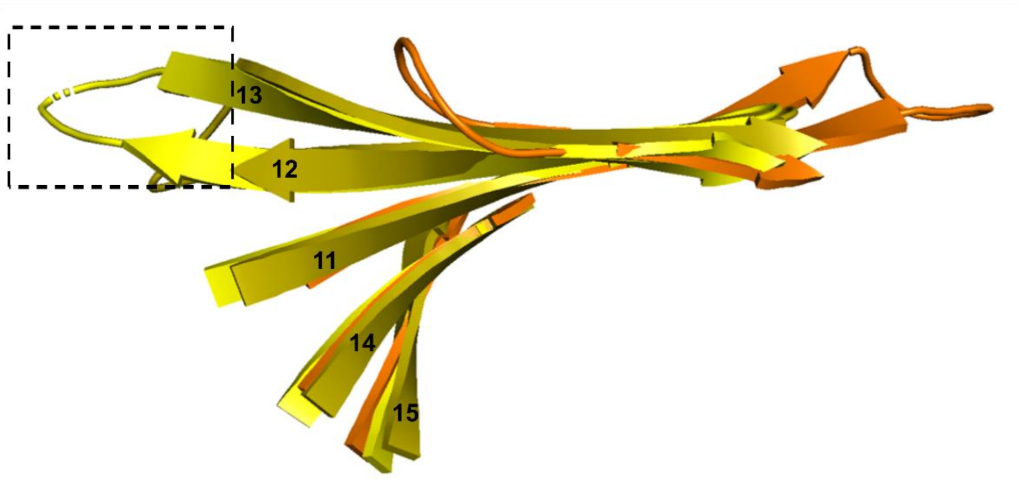

Supplementary Figure 5. Superposition of central β-sheets of RNase H domains/proteins. CaMV RT (yellow), human RNase H1 (orange) (PDB ID: 2QK9) (7), and Ty3 RNase H domain (olive) (PDB ID: 4OL8) (2). The dotted box indicates the CaMV RH domain extended β-sheets.

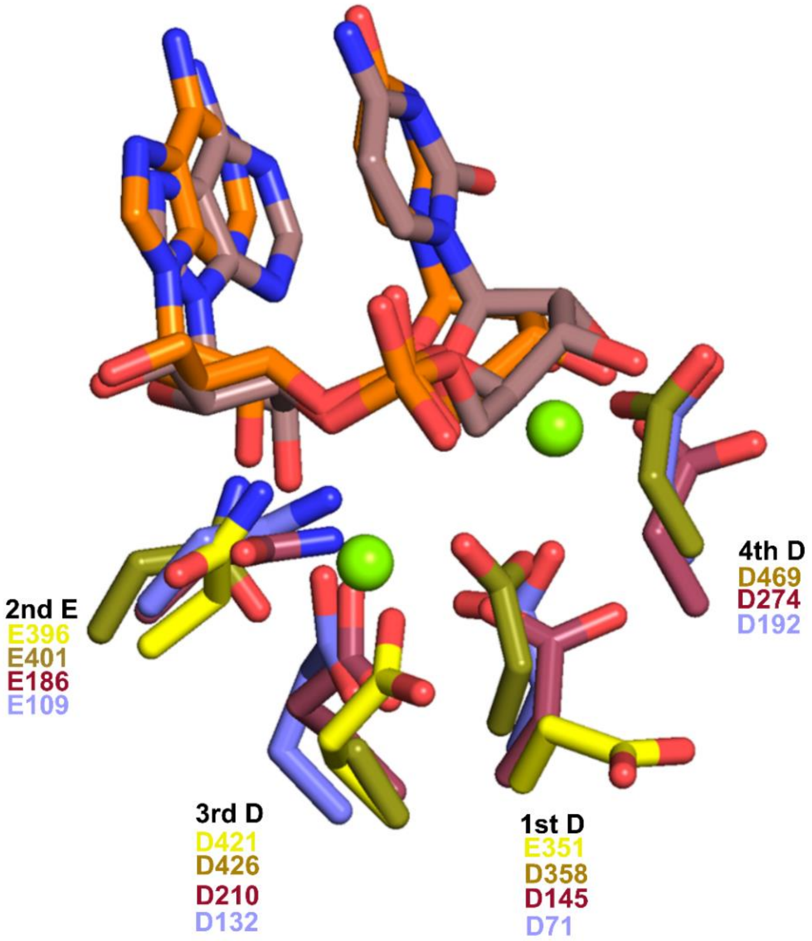

Supplementary Figure 6. Superposition of active sites of different RNase H domains. Active site residues are shown for CaMV RT (yellow), Ty3 RT (olive) (PDB ID: 4OL8) (2), human RNase H1 (raspberry) (PDB ID: 2QK9) (7), and *B. halodurans* RNase H1 (light blue) (PDB ID: 1ZBI) (8). Mg^{2+} ions from the bacterial RNase H1 structure are shown as green spheres. RNA fragments from the human and bacterial RNase H1 structures are shown in dark salmon and orange, respectively. The fourth residue of the CaMV RNase H active site, which is displaced from its canonical position is omitted for clarity.

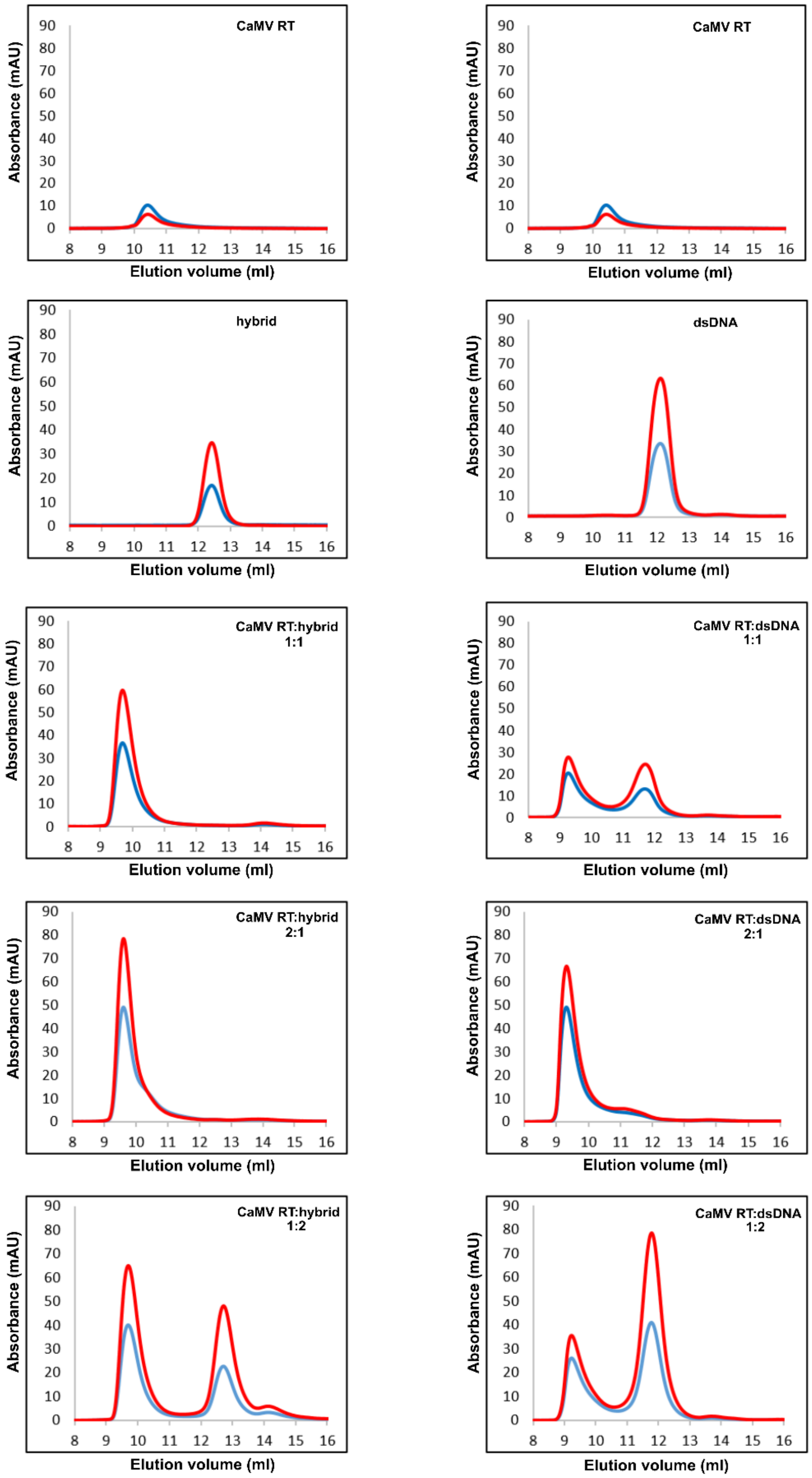

Supplementary Figure 7. **Analysis of the complex formation of CaMV RT with RNA/DNA or dsDNA by gel filtration**. Different molar ratios (1:1, 1:2, and 2:1) of protein and substrate were mixed and incubated on ice for 30 min. The complex samples were separated on a

Superdex 200 column. Elution profiles are shown. The red traces show absorbance at $\lambda = 260$ nm and blue traces at $\lambda = 280$ nm. The left column shows experiments with an RNA/DNA hybrid, and the right column shows experiments with dsDNA. Protein:substrate molar ratios are indicated in each panel.

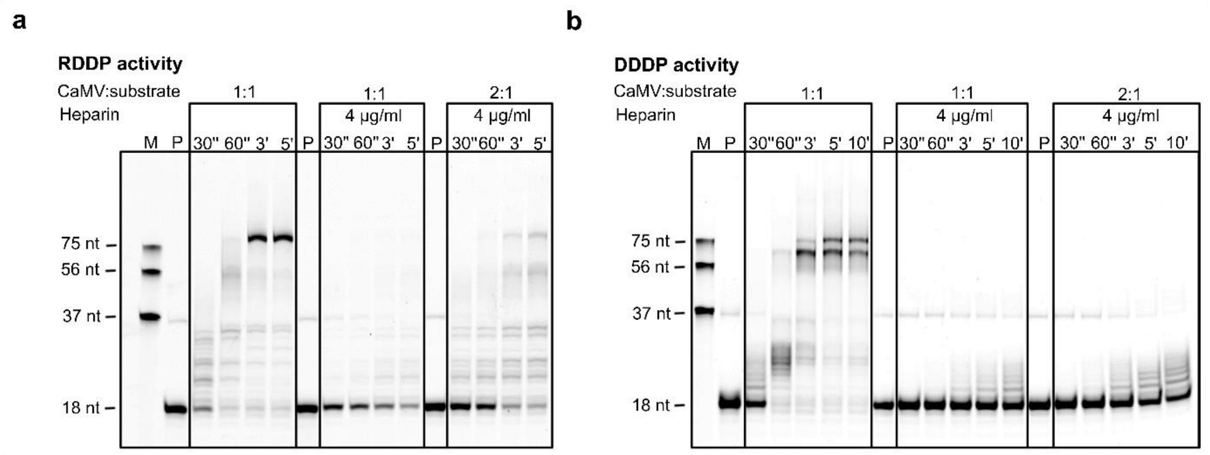

Supplementary Figure 8. Polymerase activity assay in the presence of the heparin trap. (**a**) RNA-dependent DNA polymerase (RDDP) activity assay. The CaMV RT polymerase activity assay was performed at different enzyme concentrations (100 and 200 nM) with 100 nM hybrid substrate (fluorescently labeled 18 nt DNA primer hybridized to the 3′ terminus of a 75 nt RNA template). The reaction was performed at different time intervals (30 s, 60 s, 3 min, and 5 min, marked on top of each lane) at 30°C. Selected reactions contained 4 μg/ml heparin as indicated on top of each group of lanes. The reaction products were analyzed on urea-PAGE gels with fluorescence detection. Lane M: DNA markers. Lane P: fluorescently labeled DNA primer. (**b**) DNA-dependent DNA polymerase (DDDP) activity assay. The assay was performed as in (a) with dsDNA substrate (fluorescently labeled 18 nt DNA primer hybridized to the 3' terminus of a 75 nt DNA template). The reaction was performed at different time intervals (30 s, 60 s, 3 min, 5 min and 10 min, marked on top of each lane) at 30°C.

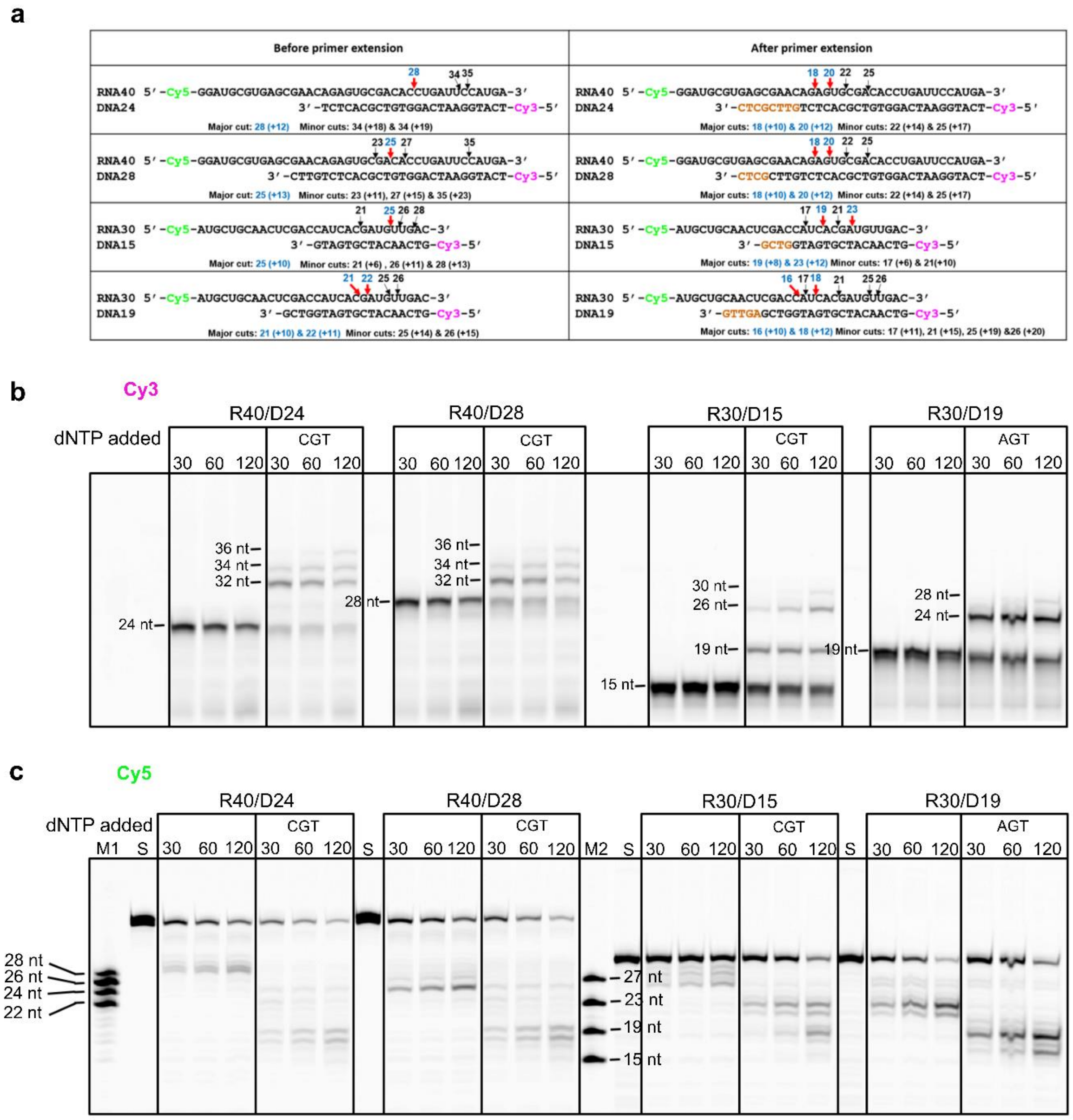

Supplementary Figure 9. **RNase H activity assays during nucleotide incorporation.** (**a**) Sequences of RNA/DNA substrates used in the experiments. Names of the substrates come from RNA (R) or DNA (D) and the length (in nt). In the left column, the cut sites for CaMV RT RNase H are marked with arrows (red arrows for major cuts and black arrows for minor cuts, product lengths in nt are given). In the right column, the cut sites for CaMV RT RNase H during nucleotide incorporation are shown. The incorporated nucleotides are shown in orange. In both columns the cuts are summarized below substrate sequence and the distance from the polymerase active site is given in parentheses. (**b** and **c**) CaMV RNase H activity assay for R40

substrate series (R40/D24, R40/D28) and R30 substrate series (R30/D15, R30/D19) with 200 nM enzyme and 100 nM substrate concentration. The reaction was performed at different time intervals (30, 60, and 120 min) at 30°C. Reaction products were analyzed on urea-PAGE scanned for fluorescence. The gels shown in (b) were scanned for Cy3 fluorescence (label on the primer strand) and in (c) for Cy5 fluorescence (label on the template strand) . Lane M: marker. Lane S: fluorescently end-labelled RNA strand. The experiments were performed three times.

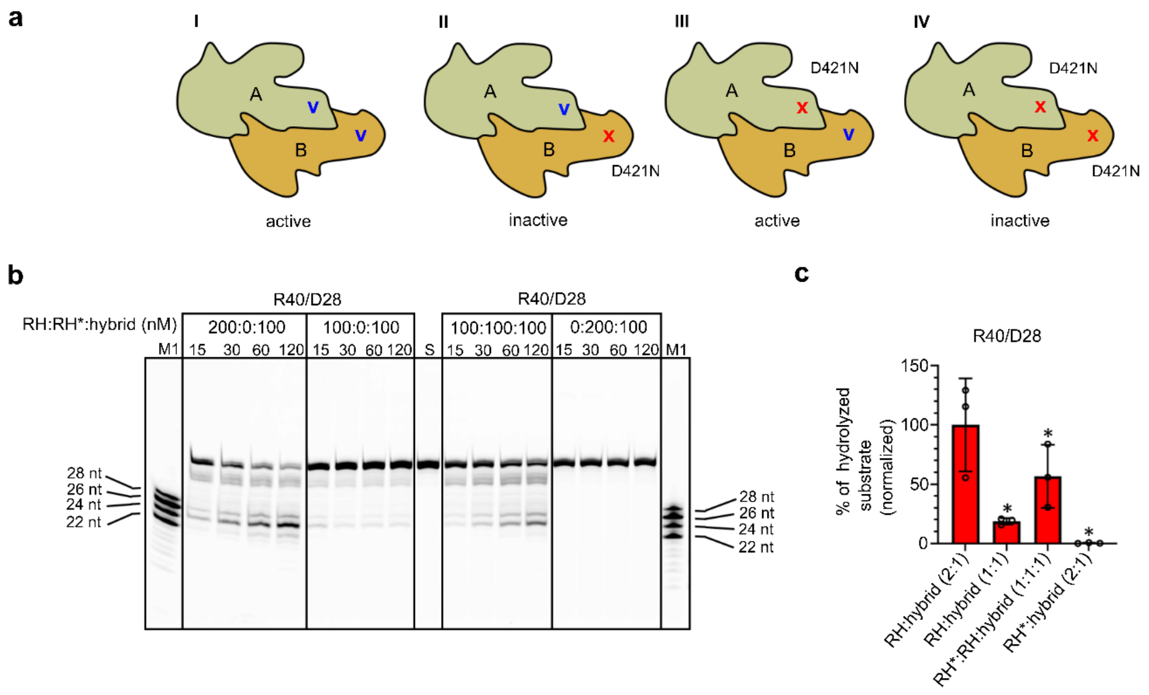

Supplementary Figure 10. RNase H activity assays of CaMV RT. (a) Cartoon representation of the possible arrangements of wt and RNase H mutant subunits in the transient CaMV RT dimer. Position of RNase H active site is indicated with blue 'V' (wt) or red 'X' (mutated). Predicted RNase H activity of each configuration is indicated below each schematic. **(b)** CaMV RNase H activity assay performed at different protein:substrate ratios using R40/D28 substrate. WT protein (RH), RNase H deficient mutant D421N (RH*) and the substrate (hybrid) were mixed at different ratios indicated on top of each group of lanes, and the reaction was performed at different time intervals (15, 30, 60, and 120 min) at 30°C. (**c**) Quantification of the data shown in (b). The amount of hydrolyzed substrate is plotted for the 15 mins time-point and different protein:substrate ratios. Percentage of hydrolyzed product value is normalized with the amount of hydrolyzed substrate in 2:1 wt protein:substrate ratio reaction set to 100%. The error bar corresponds to the standard deviation from three independent experiments and data points are shown as circles. Statistical comparisons between the 2:1 wt:hybrid RNase H activity and the activity of the other variants (1:1 wt:hybrid, 1:1:1 RH mutant:wt:hybrid, 2:1 RH mutant:hybrid) at the 15 minute time point were performed using a paired sample t-test.

Significant differences (p-value < 0.05) are indicated by one asterisk (*).

Supplementary References

1. J. Jumper, R. Evans, A. Pritzel, T. Green, M. Figurnov, O. Ronneberger, et al., Highly accurate protein structure prediction with AlphaFold, *Nature*, 596, 2021, 583–589.

2. E. Nowak, J.T. Miller, M.K. Bona, J. Studnicka, R.H. Szczepanowski, J. Jurkowski, et al., Ty3 reverse transcriptase complexed with an RNA-DNA hybrid shows structural and functional asymmetry, *Nat. Struct. Mol. Biol*., 21, 2014, 389–396.

3. L.A. Kohlstaedt, J. Wang, J.M. Friedman, P.A. Rice and T.A. Steitz, Crystal structure at 3.5 A resolution of HIV-1 reverse transcriptase complexed with aninhibitor, *Science*, 256, 1992, 1783–1790.

4. J. Pei, B.-H. Kim and N.V. Grishin, PROMALS3D: a tool for multiple protein sequence and structure alignments, *Nucleic Acids Res*., 36, 2008, 2295–2300.

5. E. Nowak, W. Potrzebowski, P.V. Konarev, J.W. Rausch, M.K. Bona, D.I. Svergun, et al., Structural analysis of monomeric retroviral reverse transcriptase in complex with an RNA/DNA hybrid, *Nucleic Acids Res*., 41, 2013, 3874–3887.

6. M. Nowacka, E. Nowak, M. Czarnocki-Cieciura, J. Jackiewicz, K. Skowronek, R.H. Szczepanowski, et al., Structures of substrate complexes of foamy viral protease-reverse transcriptase, *J. Virol*., 95, 2021, e0084821.

7. M. Nowotny, S.A. Gaidamakov, R. Ghirlando, S.M. Cerritelli, R.J. Crouch and W. Yang, Structure of human RNase H1 complexed with an RNA/DNA hybrid: insight into HIV reverse transcription, *Mol. Cell*, 28, 2007, 264–276.

8. M. Nowotny, S.A. Gaidamakov, R.J. Crouch and W. Yang, Crystal structures of RNase H bound to an RNA/DNA hybrid: substrate specificity and metal-dependent catalysis, *Cell*, 121, 2005, 1005–1016.
